# Sonic Hedgehog signaling regulates the optimal differentiation pace from early‐stage mesoderm to cardiogenic mesoderm in mice

**DOI:** 10.1111/dgd.12955

**Published:** 2025-01-09

**Authors:** Satoshi Inoue, Moe Nosetani, Yoshiro Nakajima, Shinichiro Sakaki, Hiroki Kato, Rie Saba, Naoki Takeshita, Kosuke Nishikawa, Atsuko Ueyama, Kazuhiko Matsuo, Masaki Shigeta, Daisuke Kobayashi, Tomoko Iehara, Kenta Yashiro

**Affiliations:** ^1^ Division of Anatomy and Developmental Biology, Department of Anatomy, Graduate School of Medical Science Kyoto Prefectural University of Medicine Kyoto Japan; ^2^ Department of Pediatrics, Graduate School of Medical Science Kyoto Prefectural University of Medicine Kyoto Japan; ^3^ Department of Pediatrics, Graduate School of Medicine The University of Tokyo Tokyo Japan; ^4^ Department of Radiology, Graduate School of Medical Science Kyoto Prefectural University of Medicine Kyoto Japan; ^5^ Department of Pediatrics, Graduate School of Medicine Osaka University Osaka Japan

## Abstract

*Sonic Hedgehog* (*Shh*), encoding an extracellular signaling molecule, is vital for heart development. *Shh* null mutants show congenital heart disease due to left–right asymmetry defects stemming from functional anomaly in the midline structure in mice. *Shh* signaling is also known to affect cardiomyocyte differentiation, endocardium development, and heart morphogenesis, particularly in second heart field (SHF) cardiac progenitor cells that contribute to the right ventricle, outflow tract, and parts of the atrium. Despite extensive studies, our understanding remains incomplete. Notably, *Shh* signaling is suggested to promote cardiac differentiation, while paradoxically preventing premature differentiation of SHF progenitors. In this study, we elucidate the role of *Shh* signaling in the earliest phase of cardiac differentiation. Our meta‐analysis of single‐cell RNA sequencing suggests that cardiogenic nascent mesoderm cells expressing the bHLH transcription factor *Mesp1* interact with axial mesoderm via Hh signaling. Activation of Hh signaling using a Smoothened agonist delayed or suppressed the differentiation of primitive streak cells expressing T‐box transcription factor *T* to *Mesp1* ^+^ nascent mesoderm cells both in vitro and ex vivo. Conversely, inhibition of Hh signaling by cyclopamine facilitated cardiac differentiation. The reduction of *Eomes*, an inducer of *Mesp1*, by Hh signaling appears to be the underlying mechanism of this phenomenon. Our data suggest that SHH secreted from axial mesoderm inhibits premature differentiation of *T* ^+^ cells to *Mesp1* ^+^ nascent mesoderm cells, thereby regulating the pace of cardiac differentiation. These findings enhance our comprehension of *Shh* signaling in cardiac development, underscoring its crucial role in early cardiac differentiation.

## Graphical abstract

*Sonic Hedgehog* (*Shh*) signaling is essential for cardiac development, influencing left–right asymmetry, cardiomyocyte differentiation, and heart morphogenesis, particularly in second heart field progenitors. Our meta‐analysis of single‐cell RNA sequencing data newly reveals that Shh inhibits the premature differentiation of *T* ^+^ primitive streak cells into *Mesp1* ^+^ nascent mesoderm, mediated by the suppression of Eomes. These findings shed new light on the regulatory role of Shh in early cardiac differentiation.

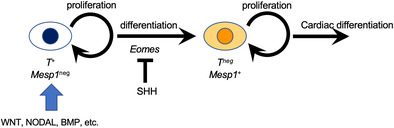

## 1 INTRODUCTION

Despite extensive studies on the signals governing cardiac specification and differentiation during embryogenesis, our understanding of the molecular mechanism underlying heart development remains incomplete. Cardiac cells, including cardiomyocytes, primarily originate from the mesoderm (Lescroart et al., 2018; Paige et al., 2015; Saga et al., 1999). Through gastrulation, epiblast cells differentiate to mesoderm cells. The earliest mesoderm cells express T‐box transcription factor *T* (also known as *Brachyury*) in the primitive streak. Among these, the cells derived from the anterior part of the primitive streak give rise to cardiogenic nascent mesoderm cells expressing the bHLH transcription factor *Mesp1*. In mouse embryos, these *Mesp1* ^+^ nascent mesoderm cells migrate anteriorly and populate cardiac progenitor cells at the anterior side of the embryo, which are marked by the cardiac genes including *Nkx2‐5*, *Isl1*, *Gata4*, and *Tbx5*.

The earliest phase of mesoderm specification is under the control of *T* and another T‐box transcription factor, *Eomes* (Costello et al., 2011; Papaioannou, 2014). Although *T* is not essential for cardiac differentiation, *Eomes* is crucial (Concepcion & Papaioannou, 2014; Costello et al., 2011). EOMES directly induces transcription of *Mesp1* in the earliest mesoderm cells expressing *T* in the primitive streak. Bmp, Wnt, and Nodal signals likely orchestrate to induce *Eomes* and then *Mesp1* among *T* ^+^ mesoderm cells (Costello et al., 2011; Tsaytler et al., 2023). However, the mechanism underlying the induction of *Eomes* remains largely unknown.

*Sonic Hedgehog* (*Shh*) encodes a signaling molecule secreted extracellularly, and is one of three homologues of *Hedgehog* (Hh) of *Drosophila melanogaster* (fruit fly) (Nusslein‐Volhard & Wieschaus, 1980). *Shh* has been shown to be involved in heart development; *Shh* null mutant mice showed heart defects due to the malfunction of the midline structure affecting left–right asymmetry (Tsukui et al., 1999; Yamamoto et al., 2003). Hh signaling is essential for the development of the endocardium, and for the structure dependent on the second heart field (SHF) cardiac progenitor cells that contribute mainly to the right ventricle, outflow tract, and part of the atrium (Dyer & Kirby, 2009; Goddeeris et al., 2007; Guzzetta et al., 2020; Hoffmann et al., 2009; Lin et al., 2006; Wong et al., 2012). Hh signaling likely promotes cardiomyocyte differentiation of P19 embryonic carcinoma cells (Clement et al., 2009; Gianakopoulos & Skerjanc, 2005). On the other hand, paradoxically, *Shh* signaling has been shown to prevent premature differentiation of SHF cardiac progenitor cells by delaying differentiation in mouse embryos, ensuring the specification of the proper number of myocardial progenitors (Rowton et al., 2021, 2022; Thomas et al., 2008). Thus, our comprehension of the role of Hh signaling in the cardiac development remains uncovered.

This study demonstrates that the Hh signal regulates the pace of differentiation from the earliest mesoderm *T* ^+^ cells to the *Mesp1* ^+^ nascent mesoderm cells. We performed meta‐analyses of single‐cell RNA sequencing data (Pijuan‐Sala et al., 2019), suggesting that cardiac cells transition from *Mesp1* ^+^ nascent mesoderm to pharyngeal mesoderm cells, and receive Hh signal from axial mesoderm. In vitro differentiation studies using mouse embryonic stem (ES) cells indicate that the activation of the Hh signal via a small molecule Smoothened agonist (SAG) attenuates the differentiation from *T* ^+^ cells to *Mesp1* ^+^ cells, whereas the inhibition of Hh via cyclopamine (CPA) promotes this differentiation. Importantly, Hh signal likely reduces *Eomes* expression. The ex vivo studies yielded results consistent with those of the in vitro study. Treatment with SAG increased the expression of *T* while reducing *Eomes* and *Mesp1* in the early mouse embryos subjected to whole embryonic culture. Conversely, CPA treatment maintained or enhanced *Eomes* and *Mesp1* expression but reduced *T*. These results collectively demonstrate that the Hh signaling regulates the timing and/or pace of differentiation from *T* ^+^ earliest mesoderm cells to *Mesp1* ^+^ nascent (cardiogenic) mesoderm cells by controlling *Eomes* induction.

## 2 MATERIALS AND METHODS

### 2.1 Animals

ICR mice were obtained from Japan SLC. All animal procedures were approved and performed according to guidelines specified by the Animal Experimentation Committee of Kyoto Prefectural University of Medicine (license nos. M2023‐169 and M2023‐174). The morning of the day on which a vaginal plug was detected after mating was determined as embryonic day (E) 0.5.

### 2.2 Whole embryonic culture

Embryos were dissected at E6.5 in Dulbecco's modified Eagle medium (DMEM) with high glucose, HEPES, and without phenol red (Cat # 21063029, ThermoFisher Scientific, USA), ensuring that the ectoplacental cone remained intact while removing Reichert's membrane. The dissected embryos were then cultured in 75% immobilized rat serum/DMEM with high glucose (Cat # 11965092, ThermoFisher Scientific, USA), with or without small molecule inhibitors, for 8 h on a 4‐well chamber slide (Cat # 192‐004, WATSON, Japan) coated with Sigmacote (Cat # SL2‐100ML, Sigma‐Aldrich, USA). Subsequently, the embryos were incubated for an additional 16 h in a centrifuge tube with rotation in a 5% CO_2_ incubator at 37°C. Following culture, the embryos were fixed in 4% paraformaldehyde (PFA) in phosphate‐buffered saline at 4°C overnight.

### 2.3 Whole mount in situ hybridization

Whole mount in situ hybridization was performed as previously described (Uehara et al., 2009). A probe of *T* was kindly gifted by H. Hamada, and a *Mesp1* probe from Y. Saga. A probe of *Eomes* was synthesized with cDNA clone (NCBI accession number: AK089817).

### 2.4 Cardiomyocyte differentiation of mouse embryonic stem cells

E14tg2a mouse ES cell line was maintained as naive ground state of pluripotency with KnockOut DMEM (10829018, ThermoFisher Scientific)/2% Fetal Bovine Serum (FBS)/10% serum replacement (191‐18375, WAKO)/0.1 mM non‐essential amino acid (NEAA) (11140050, ThermoFisher Scientific)/0.1 mM 2‐mercaptoethanol (2‐ME) (M3148, Sigma‐Aldrich)/2 mM Glutamax (35050062, ThermoFisher Scientific)/50 μg/mL of ascorbic acid (A4544, Sigma‐Aldrich)/ 3 μM CHIR99021, 1 μM of PD0325901 and 10 ng/mL mouse Leukemia Inhibitory Factor (8878‐LF/CF, R&D)/100 units/mL penicillin–streptomycin (15140122, ThermoFisher Scientific). For cardiac differentiation, 6 × 10^2^ cells were aggregated as embryoid body (EB) by hanging drop procedure in 15 μL of the first differentiation medium: Iscove's modified Dulbecco's medium (12,440,053, ThermoFisher Scientific)/15% FBS/5% serum replacement/0.1 mM NEAA/0.1 mM 2‐ME/2 mM Glutamax/50 μg/mL of ascorbic acid/100 units/mL penicillin–streptomycin. At differentiation day 2 (2 days after EB formation), EBs were seeded on the gelatin‐coated culture dish, and continuously allowed to differentiate in the first differentiation medium up to day 5. On day 5, the concentration of FBS was changed to 7.5%. Beating foci were usually visible after differentiation day 7.

### 2.5 EdU incorporation assay and cell death assay

Cells were incubated with 10 μM EdU for 30 min before assay. EdU‐positive cells were detected using the Click‐iT® EdU Alexa Fluor 647 Imaging Kit (ThermoFisher Scientific) according to the manufacturer's instructions. The antibodies used are listed in Table S1.

### 2.6 Bioinformatics analyses

#### 2.6.1 Seurat data processing

The processed data from E6.5 to E8.5 mouse embryos were obtained from ArrayExpress (Accession number: E‐MTAB‐6967) (Pijuan‐Sala et al., 2019). The data were provided as Cell Ranger output files and subsequently imported into Seurat (v4.3.0) (Hao et al., 2024), a pipeline for single‐cell RNA sequencing (scRNA‐seq) analysis. The dataset contains gene expression profiles from 86,689 cells. Cells with more than 5% mitochondrial reads and fewer than 200 detected genes were excluded. Data normalization and scaling were performed, followed by principal component analysis (PCA). For visualization, uniform manifold approximation and projection (UMAP) was used. Cluster annotations were based on the designations from the referenced article (Pijuan‐Sala et al., 2019), and marker genes for each cluster were identified using the FindAllMarkers function.

#### 2.6.2 Extraction of mesodermal subset

A sub‐dataset representing the mesodermal lineage was created by subsetting clusters including “Primitive Streak (cluster #21 in Figure S1)”, “Anterior Primitive Streak (cluster #29)”, “Notochord (cluster #20)”, “Nascent Mesoderm (cluster #15),” “Mixed Mesoderm (cluster #5)”, “ExE Mesoderm (cluster #6)”, “Pharyngeal Mesoderm (cluster #8)”, “Mesenchyme (cluster #11)”, and “Cardiomyocytes (cluster #33)”. This sub‐dataset consisted of 18,840 cells. The SCTransform function was employed to normalize and scale the data, followed by PCA and UMAP for visualization. Clustering was performed with a clustering resolution of 0.5.

#### 2.6.3 Analysis of cell–cell communication

Intercellular communication in the mesodermal subset was estimated using CellChat (v1.6.1) (Jin et al., 2021), which applies the ligand–receptor interaction database “CellChatDB”. The Seurat dataset was first converted to a CellChat dataset, and communication probabilities were calculated using the “computeCommunProb” function. Each signaling pathway was visualized using Circle and Dot plots.

#### 2.6.4 Trajectory analysis

Trajectory analysis of the mesodermal cell dataset was conducted using Monocle3 (v1.3.1) (Cao et al., 2019; Qiu, Hill, et al., 2017; Qiu, Mao, et al., 2017; Trapnell et al., 2014). Pseudotime was calculated, with the Primitive Streak cluster serving as the starting point for the trajectory. The trajectory revealed three main branches leading to differentiation into cardiomyocytes, extraembryonic mesoderm, and endoderm. We selected the branch leading to cardiomyocyte differentiation and calculated temporal changes in gene expression related to cardiomyocyte development along the pseudotime trajectory.

### 2.7 Real‐time reverse‐transcription polymerase chain reaction (RT‐PCR)

Embryos or cells were homogenized in RNAiso Plus (9108, TaKaRa). Total RNA was purified using the Direct‐zol Microprep Kit (R2060, Zymo Research) according to the manufacturer's protocol. The first strand cDNA was synthesized from 100 ng of total RNA with ReverTra Ace® qPCR RT Master Mix with gDNA Remover (FSQ301, TOYOBO) according to the manufacturer's protocol. PCR was performed with THUNDERBIRD™ *Next* SYBR® qPCR Mix (QPX201, TOYOBO) and StepOne Plus real‐time PCR system (ThermnoFisher Scientific) according to the manufacturer's protocol. Relative level of expression of each target gene was calculated using the ΔΔCT method and normalized with the data of *Gapdh* (Livak & Schmittgen, 2001). The used PCR forward (Fw) and reverse (Rv) primers for each gene are listed in Table S2.

## 3 RESULTS AND DISCUSSION

**FIGURE 1:**
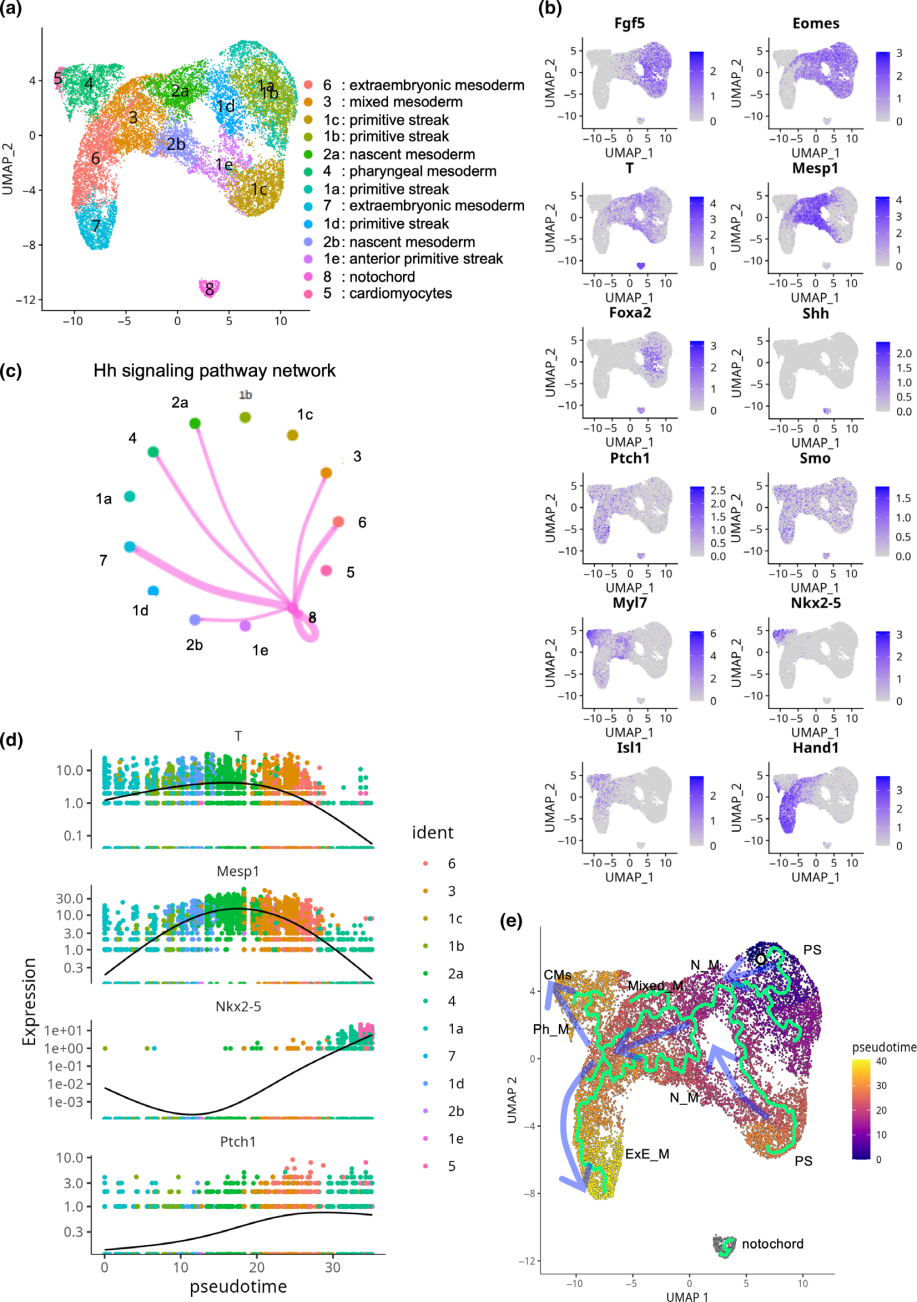
Suggested interaction between notochord (axial mesoderm) and cardiac cells via Hh signaling. (a) Uniform manifold approximation and projection (UMAP)‐based sub‐clustering of cardiac cells and notochord (axial mesoderm). Note clusters 1a, 1b, and 1d of “primitive streak” corresponded to E7.0 stage embryos, while 1c and 1e belonged to the E7.25 stage embryos as indicated in Figure S3b. (b) Marker gene expression on UMAP presented in (a). Refer to the main text for further details. (c) Intercellular communication analysis on the sub‐dataset using CellChat. CellChat analysis showed cardiac cells (clusters 2a, 2b, 3, and 4) positive for *Mesp1* interact with notochord (axial mesoderm) via Hh signaling (please also see Figure S3a). Hedgehog signaling network between clusters is visualized. See main text in detail. (d) Pseudotime analysis. *Ptch1* expression, indicative of Hh signaling, increases after *Mesp1* expression begins. *Nkx2‐5* marks the cardiac identity. (e) Cell trajectory study. The trajectory was generated using the pseudotime method on a UMAP. The semi‐transparent blue arrows indicate the direction of differentiation along the trajectory. The trajectory progresses from “primitive streak” through “nascent mesoderm”, “mixed mesoderm” and “pharyngeal mesoderm” toward “cardiomyocytes”. Additionally, “extraembryonic mesoderm” is observed to diverge from *Mesp1* ^+^ “mixed mesoderm”, supporting the validity of this in silico analysis. The two primary trajectories of differentiation from the primitive streak likely result from differences in the developmental stages: Clusters 1a, 1b, and 1d correspond to the embryonic day (E) 7.0 stage, while 1c and 1e correspond to the E7.25 stage, as indicated in Figure S3b. CMs, cardiomyocytes; ExE_M, extraembryonic mesoderm; Mixed_M, mixed mesoderm; N_M, nascent mesoderm; Ph_M, pharyngeal mesoderm; PS, primitive streak.

To examine *Shh* signaling involved in the early phase of cardiac differentiation, we performed meta‐analyses on scRNA‐seq data from the Mid‐primitive streak stage, when cardiogenic nascent mesoderm cells emerge, to the Early Headfold stage, when cardiac progenitor cells populate (Figure 1) (Downs & Davies, 1993; Pijuan‐Sala et al., 2019). Dimension reduction using UMAP classified cells into 36 clusters (Figure S1) (Becht et al., 2018). The original cluster annotations from the reference report, described as “celltype” in the metadata of the dataset, were adopted (Pijuan‐Sala et al., 2019). We identified the cluster annotated with “notochord” as axial mesoderm, because it expressed *T*, *Foxa2*, and *Shh* (Figure S1). We also identified the cluster annotated with “mixed mesoderm” as differentiating *Mesp1* ^+^ nascent mesoderm cells to lateral plate mesoderm and/or extraembryonic mesoderm. This was supported by the expression of lateral plate mesoderm markers, including *Cfc1* (*Cryptic*), *Kdr*, and *Foxf1* and extraembryonic mesoderm markers, including *Hand1* (Figures S1 and S2a) (Mahlapuu et al., 2001; Shen et al., 1997; Yamaguchi et al., 1993). In general, *Fgf5* is used to identify epiblast, whereas it was also expressed in nascent mesoderm (Figure 1b).

Next, to investigate the involvement of Hh signaling in mesodermal development contributing to heart formation, we analyzed not only the “notochord (axial mesoderm)” cluster but also potential cardiogenic lineages including “primitive streak”, “anterior primitive streak”, “nascent mesoderm (cardiogenic mesoderm)”, “mixed mesoderm”, and “cardiomyocyte” clusters for sub‐clustering analysis (Figures 1a, b and S2b). This analysis also incorporates “pharyngeal mesoderm”, a part of which is recognized as cardiogenic (Tzahor & Evans, 2011). Moreover, we decided to include “extraembryonic mesoderm”, which is not cardiogenic but is known to differentiate from *Mesp1* ^+^ cells, into the data to be analyzed. This decision was based on the hypothesis that our in silico findings would be validated if we could confirm the divergence of the extraembryonic mesoderm lineage from *Mesp1* ^+^ nascent mesoderm cells in cell trajectory analysis.

Using CellChat, our in silico analysis of cell–cell communication revealed that cardiogenic cells (clusters 2a and 2b, identified as “nascent mesoderm”, cluster 3 as “mixed mesoderm”, and cluster 4 as “pharyngeal mesoderm”) interact with the notochord (axial mesoderm) through Hh signaling (Figures 1c and S3a) (Jin et al., 2021). Consistent with previous studies, a pseudotime analysis revealed that *Mesp1* expression is significantly elevated in nascent mesoderm and then progressively declines from mixed mesoderm to cardiomyocytes via pharyngeal mesoderm, thereby substantiating the veracity of this in silico analysis (Figures 1d, e and S3) (Krup et al., 2023; Lescroart et al., 2018; Saga et al., 1996, 1999). This pseudotime analysis indicated that Hh signaling was initiated in *Mesp1* ^+^ cardiogenic nascent mesoderm and gradually intensified as the embryo developed, represented by *Ptch1* expression (Figure 1d) (Ingham & McMahon, 2001). This finding was further supported by cell trajectory analyses, which aligned with both the CellChat analysis and the temporal dynamics inferred from pseudotime analysis (Figures 1d, e and S3a, b). As expected, extraembryonic mesoderm was predicted to derive from *Mesp1* ^+^ mesoderm in this trajectory analysis, supporting the validity of this in silico study. Collectively, our in silico study suggests that Hh signaling is activated by SHH originating from the axial mesoderm when *T* ^+^ primitive streak begins to express *Mesp1*.

**FIGURE 2:**
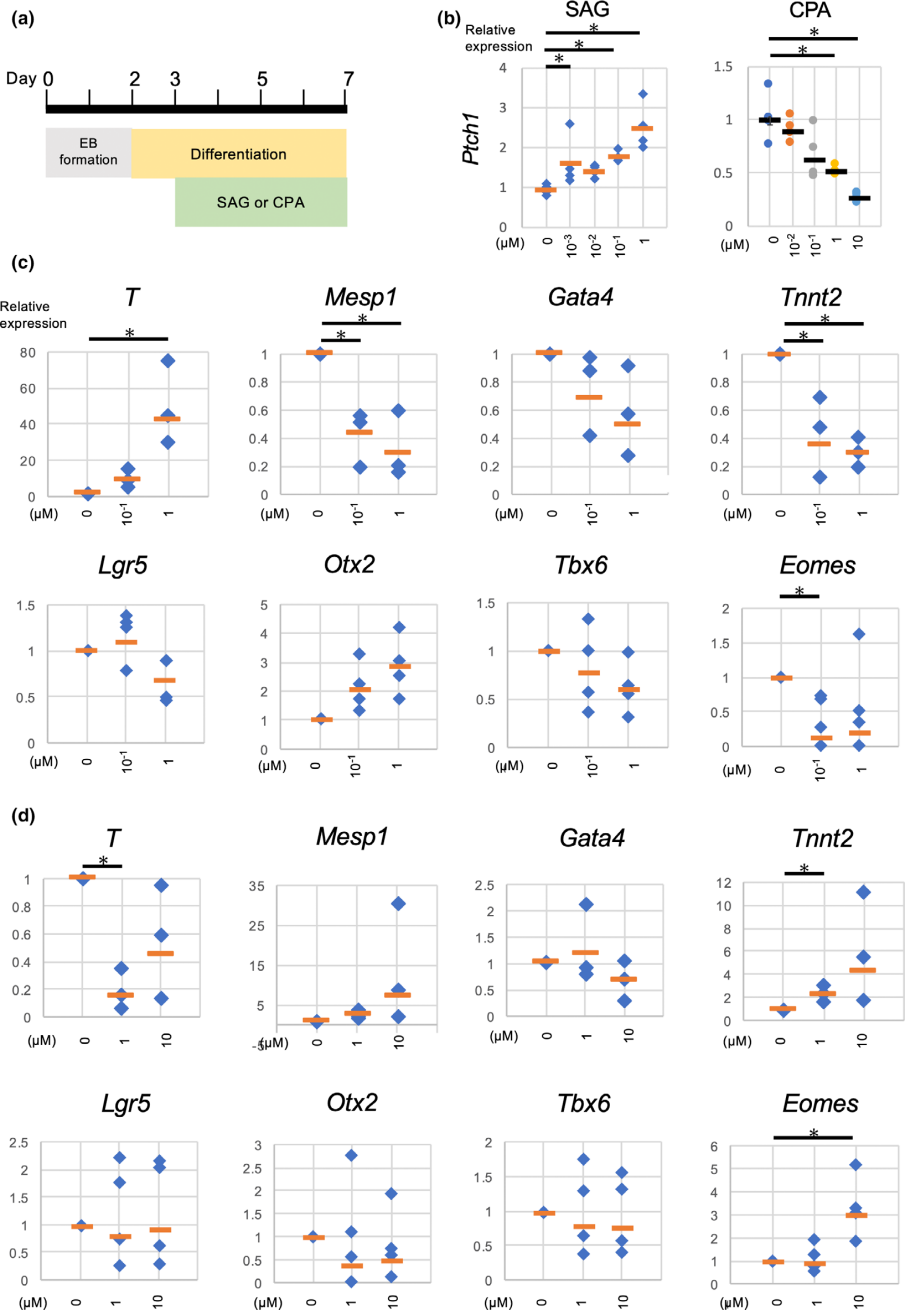
Hh signal suppress cardiac differentiation in mouse embryonic stem (ES) cell cardiac differentiation. (a) Experimental strategy of in vitro cardiac differentiation with cyclopamine (CPA) or Smoothened agonist (SAG). EB formed via hanging drop method were seeded on the culture dishes at differentiation day 2 and were left to differentiate. SAG or CPA treatment was started at differentiation day 3 when the expression of *T* peaked. The beating foci of cardiomyocytes were observed after day 7. (b) Validation of the effect of SAG and CPA on Hh signaling in cardiac differentiation of mouse ES cells. Each graph represents the result from biological triplicates or quadruplicates. The asterisk indicates *p* < .05 by Student *t* test. The orange bar represents the median value. (c) Expression level of marker genes evaluated by real time RT‐PCR in SAG treatment. All data represent biological triplicates. The asterisk indicates *p* < .05 by Student *t* test. The orange bar represents the median value. (d) Expression level of marker genes evaluated by real time RT‐PCR in CPA treatment. All data represent biological triplicates or quadruplicates. The asterisk indicates *p* < .05 by Student *t* test. The orange bar represents the median value.

The precise role of Hh signaling at the initial phase of cardiac development remains unclear. To address this, we investigated the effect of activating or inhibiting the Hh signaling pathway on cardiac differentiation using a mouse ES cells in vitro cardiomyocyte differentiation system (Figure 2a). First, we determined the appropriate concentrations of SAG to activate the Hh signal and CPA to inhibit it. Cardiac differentiation was induced through EB formation, and a gastrulation‐like event occurred at differentiation day 3, represented by the peaked expression of *T* (data not shown). We then treated EBs continuously from differentiation day 3 with SAG, CPA, or a mock treatment (solvent only), and assessed the expression of *Ptch1*, a marker of activated Hh signaling, by real‐time RT‐PCR (Figure 2b). The data revealed that 1 μM of SAG and 10 μM of CPA significantly activated and suppressed the Hh signal, respectively. This concentration was consistent with the previous reports (Fleury et al., 2016; Fu et al., 2015; Wada et al., 2009).

Based on this result, we elucidated the effect of activation or inhibition of Hh signaling on cardiac differentiation with SAG or CPA in vitro (Figure 2c). Markers were assayed at differentiation day 7: *T* for primitive streak cell formation, *Mesp1* for cardiogenic *Mesp1* ^+^ nascent mesoderm formation, *Gata4* and *Tnnt2* for cardiomyocyte differentiation, *Lgr5* for endoderm specification, *Otx2* for neural ectoderm formation, *Tbx6* for mesoderm specification, and *Eomes* for mesoendoderm formation and the induction process of *Mesp1* (Acampora et al., 2013; Chapman et al., 2003; Cheng et al., 2012; Krup et al., 2023). Firstly, we assayed whether activation of Hh signaling affects the initial phase of cardiac differentiation. SAG treatment showed suppressed or delayed differentiation of nascent mesoderm, indicated by persisted *T* expression and significantly reduced *Mesp1* and *Eomes*, along with a tendency for decreased *Gata4*. Consistent with this result, cardiomyocyte differentiation was suppressed, as evidenced by reduced *Tnnt2* expression (Figure 2c) and fewer beating foci in number (data not shown). The specification and differentiation of endoderm, mesoderm, and neural ectoderm were unlikely to be affected, as indicated by the expressions of *Lgr5*, *Otx2*, and *Tbx6*, which were not significantly altered. Hence, Hh signaling appears to inhibit the differentiation of *T* ^+^ primitive streak cells into *Mesp1* ^+^ cardiogenic nascent mesoderm. Next, we assayed the effect of the inhibition of Hh signaling on cardiac differentiation via CPA treatment (Figure 2d). At differentiation day 7, we observed the opposite of the case of Hh signaling activation. *T* was significantly decreased. Although *Mesp1* showed its tendency to increase, *Eomes* was significantly increased. These drug‐induced changes in *T* were likely due to altered transcription rather than changes in cell number (Figure S4). Consistent with this result, *Tnnt2* was significantly elevated (Figure 2d), and more beating foci were found on the culture dishes (data not shown). The specification and differentiation of endoderm, mesoderm, and neural ectoderm were unlikely to be affected.

**FIGURE 3:**
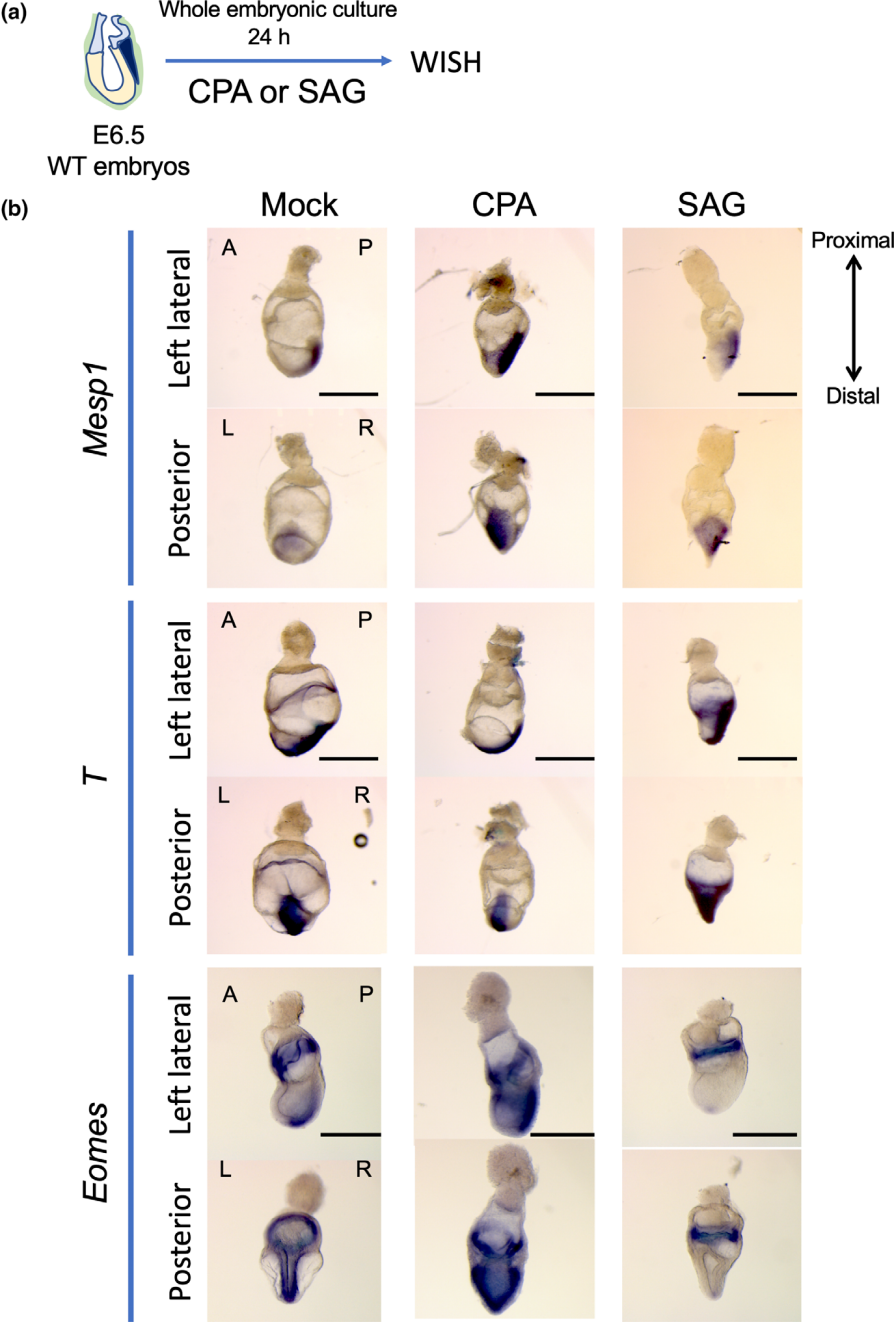
Hh signaling prevents *T* ^+^ primitive streak cells from differentiating. (a) The strategy of whole embryonic culture with inhibiting or activating Hh signaling. The dissected E6.5 mouse embryos were subjected to whole embryo culture with or without cyclopamine (CPA) or Smoothened agonist (SAG), followed by whole mount in situ hybridization (WISH). (b) WISH for *Mesp1*, *T*, and *Eomes* on the embryos after whole embryonic culture with SAG or CPA. The data shown are representative of data from three individuals for *Mesp1* and *T*, all showing the same results. Note that treatment with CPA increases *Mesp1* expression and decreases *T* expression, while SAG treatment shows exactly the opposite. For *Eomes*, the data shown are representative of six Mock samples, four of five SAG samples, and five of six CPA samples, demonstrating consistent results. Please also see Figure S5. Scale bar, 500 μm. A, anterior; L, left; P, posterior; R, right.

**FIGURE 4:**
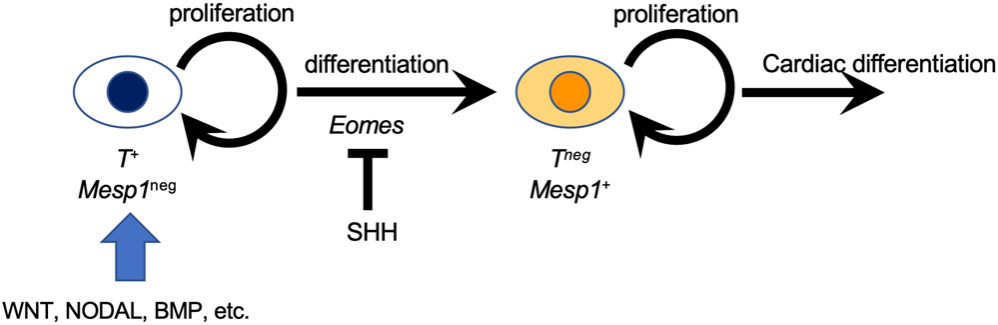
A proposed model of the role of Hh signaling in earliest cardiac differentiation. The *Shh* signaling from the axial mesoderm is likely to prevent premature differentiation from *T* ^+^ primitive streak cells to *Mesp1* ^+^ cardiogenic mesoderm cells by regulating *Eomes*. Please see the main text for further details.

Finally, we examined the effect of Hh activation or inhibition on the differentiation of *T* ^+^ primitive streak cells to *Mesp1* ^+^ cardiogenic mesoderm cells in early mouse embryos using whole embryonic culture system (Figures 3, S5 and S6). E6.5 mouse embryos were isolated and cultured for 24 h with or without SAG or CPA (Figures 2b and S6a). SAG treatment led to persistent or activated *T* expression and reduced *Eomes* and *Mesp1* expression (Figures 3b and S5). Conversely, CPA treatment led to the activation of both *Eomes* and *Mesp1*, accompanied by a reduction of *T* expression. These results were consistent with the findings observed in the in vitro ES cell differentiation experiments (Figure 2). We concluded that Hh signaling governs the differentiation of *T* ^+^ primitive streak cells to *Mesp1* ^+^ nascent mesoderm cells (Figure 4).

The molecular function of Hh signal might be to control *Eomes* expression at the earliest phase of the cardiac differentiation (Figure 4). Our data revealed that increased or decreased Hh signal suppressed or activated *Eomes* expression, respectively (Figures 2c,d and 3b). Given the evidence that *Eomes* directly induces *Mesp1* in the primitive streak (Costello et al., 2011) as well as that the most anterior part of the primitive streak gives rise to cardiac cells in the mid‐primitive streak stage (Kinder et al., 1999; Tam et al., 1997), the spatial relationship of axial mesoderm expressing *Shh* and the future cardiac cells seems rational. However, it remains unknown how Hh signaling influences *Eomes* expression.

What is the biological significance of Hh signaling in regulating cardiac differentiation at this earliest phase of development? It is likely that *Shh* modulates the differentiation speed of *T* ^+^ primitive streak cells into *Mesp1* ^+^ cardiogenic mesoderm cells to ensure proper timing and maintain an adequate pool of cardiac cells. Indeed, both *Shh* and *Smo* null mutants exhibit smaller underdeveloped hearts along with other malformations linked to laterality defects (Tsukui et al., 1999; Zhang et al., 2001). Similarly, conditional mutant mice with *Smo* specifically knocked out in *Mesp1* ^+^ cardiogenic mesoderm also show smaller underdeveloped hearts at E9.5 (Guzzetta et al., 2020). This suggests that the pool of cardiac cells was diminished as a result of the immature differentiation of *Mesp1* ^+^ cardiogenic mesoderm cells in the absence of *Shh* signaling. Conversely, the activation of Hh signaling through the loss of *Ptch1* function in mice also results in smaller embryonic hearts (Goodrich et al., 1997), implying that excessive Hh signaling may reduce the number of *Mesp1* ^+^ cardiogenic mesoderm cells by inhibiting the differentiation of *T* ^+^ primitive streak cells. Given the following evidence that SHH maintains proliferative states and delays differentiation at later stages of heart development (Dyer & Kirby, 2009; Fernandes‐Silva et al., 2017; Goddeeris et al., 2007, 2008; Placzek & Briscoe, 2018; Rowton et al., 2022; Tickle & Towers, 2017; Zhou et al., 2017), it is reasonable to propose that Hh signaling, through the axial mesoderm, regulates the pace of early cardiac differentiation from *T* ^+^ primitive streak cells to *Mesp1* ^+^ cardiogenic mesoderm cells.

Despite advances in our understanding, the reasons why cardiac differentiation of P19 embryonic carcinoma cells and zebrafish cardiac progenitors is promoted by Hh signaling remain unclear (Gianakopoulos & Skerjanc, 2005; Thomas et al., 2008). In addition, during the formation of the atrial septum, a subset of SHF cardiac progenitor cells is known to differentiate into atrial cardiomyocytes in response to *Shh* signaling (Goddeeris et al., 2008; Hoffmann et al., 2009; Zhou et al., 2017). This evidence strongly suggests that the function of *Shh* signaling among cardiac cells may be context‐dependent, varying depending on the specific stage of development. To address these research questions and ascertain whether Shh prevents premature cardiac differentiation (Figure S6b), further studies are needed.

## AUTHOR CONTRIBUTIONS

KY conceived and designed the study. KY, SI, and YN planned the experiments. SI performed bioinformatics analyses, and HK, SS, and NT assisted bioinformatics analyses. SI, MN, YN, MS, KM, and KY performed the experiments. KN, AU, DK, and TI contributed to the analyses and interpretation of data. KY and SI wrote the manuscript. All authors discussed the results and commented on the manuscript.

## CONFLICT OF INTEREST STATEMENT

The authors declare no competing interests.

## Supporting information


**Figure S1.** Meta‐analysis of single‐cell (sc) RNA‐seq data from mouse embryos spanning the gastrula to the Early Headfold stage. (a) UMAP visualization of scRNA‐seq data, originally generated by Pijuan‐Sala et al. and deposited under accession number E‐MTAB‐6967 (Pijuan‐Sala et al., 2019). UMAP identified 37 clusters, each annotated according to previous report (Hao et al., 2024; Pijuan‐Sala et al., 2019). Notably, the *T* ^+^/*Foxa1* ^+^ cluster, originally annotated as “notochord”, reinterpreted as axial mesoderm expressing *Shh*. Additionally, we identified “mixed mesoderm” population as an intermediate state in the differentiation from *Mesp1* ^+^ cardiogenic nascent mesoderm to lateral plate mesoderm and/or extraembryonic mesoderm. The clusters of “Primitive streak (cluster 21 in Figure S1)”, “Anterior primitive streak (cluster 29)”, “Notochord (cluster 20)”, “Nascent mesoderm (cluster 15)”, “Mixed mesoderm (cluster 5)”, “Extraembryonic mesoderm (cluster 6)”, “Pharyngeal mesoderm (cluster 8)”, and “Cardiomyocytes (cluster 33)” were subjected to sub‐clustering shown in Figure 1. Each cluster was annotated as followings according to the reference study: 1, Extraembryonic endoderm; 2, Rostral neuroectoderm; 3, Blood progenitors 2; 4, Extraembryonic ectoderm; 5, Mixed mesoderm; 6, Extraembryonic mesoderm; 7, Intermediate mesoderm; 8, Pharyngeal mesoderm; 9, Caudal epiblast; 10, Primordial germ cells; 11, Mesenchyme; 12, Hematoendothelial progenitors; 13, Blood progenitors 1; 14, Surface ectoderm; 15, Nascent mesoderm; 16, Gut; 17, Paraxial mesoderm; 18, Caudal neuroectoderm; 19, Epiblast; 20, Notochord; 21, Primitive streak; 22, Somitic mesoderm; 23, Visceral endoderm; 24, Caudal mesoderm；25, Erythroid 1; 26, Definitive endoderm; 27, Parietal endoderm; 28, Allantois; 29, Anterior primitive streak; 30, Endothelium; 31, Forebrain/Midbrain/Hindbrain; 32, Spinal cord; 33, Cardiomyocytes; 34, Erythroid 2; 35, Neuromesodermal progenitors; 36, Erythroid 3. Further details are provided in the main text. (b) The principal marker genes used to annotate each cluster in (a).

**Figure S2.** Features of “mixed mesoderm”. (a) Genes expressed in “mixed mesoderm” cluster in Figure S1a. Note that mixed mesoderm expressed *Cfc1* (*Cryptic*), *Kdr*, and *Mesp1*, representing that it is differentiating lateral plate mesoderm cells. (b) The principal marker genes used to annotate each cluster in sub‐clustering represented in Figure 1a. Based on these markers, clusters 1a, 1b, 1c, 1d, and 1e in Figure 1a were identified as the “primitive streak” and “anterior primitive streak”. Clusters 2a and 2b were classified as “nascent mesoderm”, cluster 3 as “mixed mesoderm”, cluster 4 as “pharyngeal mesoderm”, cluster 5 as “cardiomyocytes”, clusters 6 and 7 as “extraembryonic mesoderm”, and cluster 8 as “notochord”. The markers *Fgf8*, *Mesp1*, *Kdr/Mesp1*, *Id2/Mef2*, *Nkx2‐5*, *Hand1*, and *Foxa2* identify the “primitive streak”. “nascent mesoderm”. “mixed mesoderm”. “pharyngeal mesoderm”. “cardiomyocytes”. “extraembryonic mesoderm”. and “notochord”. respectively.

**Figure S3.** Supporting data related to Figure 1. (a) Intercellular communication analysis was performed on the sub‐dataset using CellChat, as shown in Figure 1c. The CellChat analysis revealed that cardiac cells (clusters 2a, 2b, 3, and 4), which are positive for *Mesp1*, interact with the notochord (axial mesoderm) via Hedgehog (Hh) signaling. Refer to the main text for further details. (b) The embryonic stages corresponding to the clusters represented in Figure 1a, e are noted. The identification of two major primitive streak clusters is likely due to differences in the developmental stages.

**Figure S4.** The number of T‐expressing cells is not significantly altered by CPA or SAG treatment. (a) The ratio of apoptotic cells among T‐positive cells. The proportion of apoptotic cells among T‐positive cells differentiated from mouse ES cells was evaluated using immunofluorescence staining for cleaved Caspase‐3 and T on differentiation day 4, as presented in the box plot. These results are based on biological triplicate experiments. CPA treatment did not affect the proportion of apoptotic cells. In contrast, SAG treatment significantly increased apoptosis, despite a marked increase in *T* expression observed both in vitro and ex vivo with SAG treatment (Figures 2 and 3). Asterisk indicates statistical significance as *p* < .05. n.s., not significant. (b) The ratio of proliferative cells among T‐positive cells. The proportion of proliferative cells among T‐positive cells from mouse ES cells was assessed using the EdU incorporation assay and immunofluorescence staining for T on differentiation day 4, as shown in the box plot. These findings are based on results from biological triplicate experiments. CPA treatment did not significantly affect the proportion of proliferative cells. Conversely, SAG treatment markedly reduced the proportion of proliferative cells, despite a significant increase in *T* expression observed both in vitro and ex vivo with SAG treatment (Figures 2 and 3). Asterisk indicates statistical significance as *p* < .05. n.s., not significant. (c) The ratio of T‐positive cells among all cells. The proportion of T‐positive cells among the total cell population was assessed via immunofluorescence staining for T on differentiation day 4 and is depicted as a box plot. Notably, SAG treatment did not significantly affect the proportion of T‐positive cells, despite inducing apoptosis (a) and suppressing proliferation (b). While the inhibition of differentiation could potentially increase the number of T‐positive cells, the observed induction of apoptosis and suppression of proliferation (as shown in a and b) likely counteracted this effect, resulting in no significant change in the proportion of T‐positive cells following SAG treatment. n.s., not significant.

**Figure S5.** The expression of *Eomes* in the embryos treated by SAG or CPA, not shown in Figure 3. For each group, upper column shows left lateral view, whereas the lower column shows posterior view. Data from the remaining five control embryos, four embryos treated with SAG, and five embryos treated with CPA, are presented here. Interestingly, the expression of *Eomes* in the extraembryonic tissue (Hancock et al., 1999) seemed to be not affected by altered Shh signaling. Asterisks indicate embryos with *Eomes* expression levels comparable to those of the control group. Scale bar, 250 μm.

**Figure S6.** The effect of SAG and CPA on Shh signal and cardiac differentiation in ex vivo embryos. The orange bar represents the median value. Embryos cultured for 24 h with SAG or CPA treatment were analyzed by real‐time RT‐PCR to assess the expression of *Ptch1*, a marker of Shh signaling (a), and *Myl7*, a sarcomeric gene indicative of cardiomyocyte differentiation (b). For *Ptch1*, the data include biological triplicates for the Mock treatment, quintuplets for SAG, and triplicates for CPA. No statistically significant differences were observed in the expression levels of *Ptch1* and *Myl7* in both SAG and CPA treatments. However, *Ptch1* expression exhibited a trend of upregulation with SAG treatment and downregulation with CPA treatment, suggesting that SAG activates Shh signaling, while CPA suppresses it ex vivo (a). For *Myl7*, SAG treatment showed a trend toward decreased expression (b). Unfortunately, premature differentiation by CPA or suppressed cardiac differentiation by SAG were not clearly confirmed in these experiments. The lack of significant differences may be attributed to signals from cardiac cells being obscured by the presence of numerous non‐cardiac cells in the embryos. Alternatively, this could result from the challenge of harvesting mouse embryos at precisely the same developmental stage, leading to discrepancies in developmental stages at the start and end of the culture, which could contribute to high individual variation in drug effects.

**Table S1.** Used antibodies.

**Table S2.** The used PCR primers.

## References

[dgd12955-bib-0001] Acampora, D. , Di Giovannantonio, L. G. , & Simeone, A. (2013). Otx2 is an intrinsic determinant of the embryonic stem cell state and is required for transition to a stable epiblast stem cell condition. Development, 140, 43–55. 23154415 10.1242/dev.085290

[dgd12955-bib-0002] Becht, E. , McInnes, L. , Healy, J. , Dutertre, C. A. , Kwok, I. , Ng, L. , Ginhoux, F. , & Newell, E. (2018). Dimensionality reduction for visualizing single‐cell data using UMAP. Nature Biotechnology, 37, 38–44. 10.1038/nbt.4314 30531897

[dgd12955-bib-0003] Cao, J. , Spielmann, M. , Qiu, X. , Huang, X. , Ibrahim, D. M. , Hill, A. J. , Zhang, F. , Mundlos, S. , Christiansen, L. , Steemers, F. J. , Trapnell, C. , & Shendure, J. (2019). The single‐cell transcriptional landscape of mammalian organogenesis. Nature, 566, 496–502. 30787437 10.1038/s41586-019-0969-x PMC6434952

[dgd12955-bib-0004] Chapman, D. L. , Cooper‐Morgan, A. , Harrelson, Z. , & Papaioannou, V. E. (2003). Critical role for Tbx6 in mesoderm specification in the mouse embryo. Mechanisms of Development, 120, 837–847. 12915233 10.1016/s0925-4773(03)00066-2

[dgd12955-bib-0005] Cheng, X. , Ying, L. , Lu, L. , Galvão, A. M. , Mills, J. A. , Lin, H. C. , Kotton, D. N. , Shen, S. S. , Nostro, M. C. , Choi, J. K. , Weiss, M. J. , French, D. L. , & Gadue, P. (2012). Self‐renewing endodermal progenitor lines generated from human pluripotent stem cells. Cell Stem Cell, 10, 371–384. 22482503 10.1016/j.stem.2012.02.024 PMC3580854

[dgd12955-bib-0006] Clement, C. A. , Kristensen, S. G. , Møllgård, K. , Pazour, G. , Yoder, B. , Larsen, L. , & Christensen, S. (2009). The primary cilium coordinates early cardiogenesis and hedgehog signaling in cardiomyocyte differentiation. Journal of Cell Science, 122, 3070–3082. 19654211 10.1242/jcs.049676 PMC2729259

[dgd12955-bib-0007] Concepcion, D. , & Papaioannou, V. E. (2014). Nature and extent of left/right axis defects in T(Wis) /T(Wis) mutant mouse embryos. Developmental Dynamics, 243, 1046–1053. 24801048 10.1002/dvdy.24144 PMC4238287

[dgd12955-bib-0008] Costello, I. , Pimeisl, I. M. , Dräger, S. , Bikoff, E. K. , Robertson, E. J. , & Arnold, S. J. (2011). The T‐box transcription factor Eomesodermin acts upstream of Mesp1 to specify cardiac mesoderm during mouse gastrulation. Nature Cell Biology, 13, 1084–1091. 21822279 10.1038/ncb2304 PMC4531310

[dgd12955-bib-0009] Downs, K. M. , & Davies, T. (1993). Staging of gastrulating mouse embryos by morphological landmarks in the dissecting microscope. Development, 118, 1255–1266. 8269852 10.1242/dev.118.4.1255

[dgd12955-bib-0010] Dyer, L. A. , & Kirby, M. L. (2009). Sonic hedgehog maintains proliferation in secondary heart field progenitors and is required for normal arterial pole formation. Developmental Biology, 330, 305–317. 19361493 10.1016/j.ydbio.2009.03.028 PMC2810612

[dgd12955-bib-0011] Fernandes‐Silva, H. , Correia‐Pinto, J. , & Moura, R. S. (2017). Canonical sonic hedgehog signaling in early lung development. Journal of Developmental Biology, 5, 3. 10.3390/jdb5010003 PMC5831770 29615561

[dgd12955-bib-0012] Fleury, A. , Hoch, L. , Martinez, M. C. , Faure, H. , Taddei, M. , Petricci, E. , Manetti, F. , Girard, N. , Mann, A. , Jacques, C. , Larghero, J. , Ruat, M. , Andriantsitohaina, R. , & le Lay, S. (2016). Hedgehog associated to microparticles inhibits adipocyte differentiation via a non‐canonical pathway. Scientific Reports, 6, 23479. 27010359 10.1038/srep23479 PMC4806302

[dgd12955-bib-0013] Fu, X. , Zhu, M. J. , Dodson, M. V. , & Du, M. (2015). AMP‐activated protein kinase stimulates Warburg‐like glycolysis and activation of satellite cells during muscle regeneration. The Journal of Biological Chemistry, 290, 26445–26456. 26370082 10.1074/jbc.M115.665232 PMC4646303

[dgd12955-bib-0014] Gianakopoulos, P. J. , & Skerjanc, I. S. (2005). Hedgehog signaling induces cardiomyogenesis in P19 cells. The Journal of Biological Chemistry, 280, 21022–21028. 15793308 10.1074/jbc.M502977200

[dgd12955-bib-0015] Goddeeris, M. M. , Rho, S. , Petiet, A. , Davenport, C. L. , Johnson, G. A. , Meyers, E. N. , & Klingensmith, J. (2008). Intracardiac septation requires hedgehog‐dependent cellular contributions from outside the heart. Development, 135, 1887–1895. 18441277 10.1242/dev.016147 PMC2746050

[dgd12955-bib-0016] Goddeeris, M. M. , Schwartz, R. , Klingensmith, J. , & Meyers, E. N. (2007). Independent requirements for hedgehog signaling by both the anterior heart field and neural crest cells for outflow tract development. Development, 134, 1593–1604. 17344228 10.1242/dev.02824

[dgd12955-bib-0017] Goodrich, L. V. , Milenkovic, L. , Higgins, K. M. , & Scott, M. P. (1997). Altered neural cell fates and medulloblastoma in mouse patched mutants. Science, 277, 1109–1113. 9262482 10.1126/science.277.5329.1109

[dgd12955-bib-0018] Guzzetta, A. , Koska, M. , Rowton, M. , Sullivan, K. R. , Jacobs‐Li, J. , Kweon, J. , Hidalgo, H. , Eckart, H. , Hoffmann, A. D. , Back, R. , Lozano, S. , Moon, A. M. , Basu, A. , Bressan, M. , Pott, S. , & Moskowitz, I. P. (2020). Hedgehog‐FGF signaling axis patterns anterior mesoderm during gastrulation. Proceedings of the National Academy of Sciences of the United States of America, 117, 15712–15723. 32561646 10.1073/pnas.1914167117 PMC7354932

[dgd12955-bib-0019] Hancock, S. N. , Agulnik, S. I. , Silver, L. M. , & Papaioannou, V. E. (1999). Mapping and expression analysis of the mouse ortholog of xenopus Eomesodermin. Mechanisms of Development, 81, 205–208. 10330501 10.1016/s0925-4773(98)00244-5

[dgd12955-bib-0020] Hao, Y. , Stuart, T. , Kowalski, M. H. , Choudhary, S. , Hoffman, P. , Hartman, A. , Srivastava, A. , Molla, G. , Madad, S. , Fernandez‐Granda, C. , & Satija, R. (2024). Dictionary learning for integrative, multimodal and scalable single‐cell analysis. Nature Biotechnology, 42, 293–304. 10.1038/s41587-023-01767-y PMC10928517 37231261

[dgd12955-bib-0021] Hoffmann, A. D. , Peterson, M. A. , Friedland‐Little, J. M. , Anderson, S. A. , & Moskowitz, I. P. (2009). Sonic hedgehog is required in pulmonary endoderm for atrial septation. Development, 136, 1761–1770. 19369393 10.1242/dev.034157 PMC2673765

[dgd12955-bib-0022] Ingham, P. W. , & Mcmahon, A. P. (2001). Hedgehog signaling in animal development: Paradigms and principles. Genes & Development, 15, 3059–3087. 11731473 10.1101/gad.938601

[dgd12955-bib-0023] Jin, S. , Guerrero‐Juarez, C. F. , Zhang, L. , Chang, I. , Ramos, R. , Kuan, C. H. , Myung, P. , Plikus, M. V. , & Nie, Q. (2021). Inference and analysis of cell‐cell communication using CellChat. Nature Communications, 12, 1088. 10.1038/s41467-021-21246-9 PMC7889871 33597522

[dgd12955-bib-0024] Kinder, S. J. , Tsang, T. E. , Quinlan, G. A. , Hadjantonakis, A. K. , Nagy, A. , & Tam, P. P. (1999). The orderly allocation of mesodermal cells to the extraembryonic structures and the anteroposterior axis during gastrulation of the mouse embryo. Development, 126, 4691–4701. 10518487 10.1242/dev.126.21.4691

[dgd12955-bib-0025] Krup, A. L. , Winchester, S. , Ranade, S. S. , et al. (2023). A Mesp1‐dependent developmental breakpoint in transcriptional and epigenomic specification of early cardiac precursors. Development, 150, dev201229. 10.1242/dev.201229 PMC10259516 36994838

[dgd12955-bib-0026] Lescroart, F. , Wang, X. , Lin, X. , Swedlund, B. , Gargouri, S. , Sànchez‐Dànes, A. , Moignard, V. , Dubois, C. , Paulissen, C. , Kinston, S. , Göttgens, B. , & Blanpain, C. (2018). Defining the earliest step of cardiovascular lineage segregation by single‐cell RNA‐seq. Science, 359, 1177–1181. 29371425 10.1126/science.aao4174 PMC6556615

[dgd12955-bib-0027] Lin, L. , Bu, L. , Cai, C. L. , Zhang, X. , & Evans, S. (2006). Isl1 is upstream of sonic hedgehog in a pathway required for cardiac morphogenesis. Developmental Biology, 295, 756–763. 16687132 10.1016/j.ydbio.2006.03.053

[dgd12955-bib-0028] Livak, K. J. , & Schmittgen, T. D. (2001). Analysis of relative gene expression data using real‐time quantitative PCR and the 2(‐Delta Delta C(T)) method. Methods, 25, 402–408. 11846609 10.1006/meth.2001.1262

[dgd12955-bib-0029] Mahlapuu, M. , Ormestad, M. , Enerback, S. , & Carlsson, P. (2001). The forkhead transcription factor Foxf1 is required for differentiation of extra‐embryonic and lateral plate mesoderm. Development, 128, 155–166. 11124112 10.1242/dev.128.2.155

[dgd12955-bib-0030] Nusslein‐Volhard, C. , & Wieschaus, E. (1980). Mutations affecting segment number and polarity in drosophila. Nature, 287, 795–801. 6776413 10.1038/287795a0

[dgd12955-bib-0031] Paige, S. L. , Plonowska, K. , Xu, A. , & Wu, S. M. (2015). Molecular regulation of cardiomyocyte differentiation. Circulation Research, 116, 341–353. 25593278 10.1161/CIRCRESAHA.116.302752 PMC4299877

[dgd12955-bib-0032] Papaioannou, V. E. (2014). The T‐box gene family: Emerging roles in development, stem cells and cancer. Development, 141, 3819–3833. 25294936 10.1242/dev.104471 PMC4197708

[dgd12955-bib-0033] Pijuan‐Sala, B. , Griffiths, J. A. , Guibentif, C. , Hiscock, T. W. , Jawaid, W. , Calero‐Nieto, F. J. , Mulas, C. , Ibarra‐Soria, X. , Tyser, R. C. V. , Ho, D. L. L. , Reik, W. , Srinivas, S. , Simons, B. D. , Nichols, J. , Marioni, J. C. , & Göttgens, B. (2019). A single‐cell molecular map of mouse gastrulation and early organogenesis. Nature, 566, 490–495. 30787436 10.1038/s41586-019-0933-9 PMC6522369

[dgd12955-bib-0034] Placzek, M. , & Briscoe, J. (2018). Sonic hedgehog in vertebrate neural tube development. The International Journal of Developmental Biology, 62, 225–234. 29616731 10.1387/ijdb.170293jb

[dgd12955-bib-0035] Qiu, X. , Hill, A. , Packer, J. , Lin, D. , Ma, Y. A. , & Trapnell, C. (2017). Single‐cell mRNA quantification and differential analysis with census. Nature Methods, 14, 309–315. 28114287 10.1038/nmeth.4150 PMC5330805

[dgd12955-bib-0036] Qiu, X. , Mao, Q. , Tang, Y. , Wang, L. , Chawla, R. , Pliner, H. A. , & Trapnell, C. (2017). Reversed graph embedding resolves complex single‐cell trajectories. Nature Methods, 14, 979–982. 28825705 10.1038/nmeth.4402 PMC5764547

[dgd12955-bib-0037] Rowton, M. , Guzzetta, A. , Rydeen, A. B. , & Moskowitz, I. P. (2021). Control of cardiomyocyte differentiation timing by intercellular signaling pathways. Seminars in Cell & Developmental Biology, 118, 94–106. 34144893 10.1016/j.semcdb.2021.06.002 PMC8968240

[dgd12955-bib-0038] Rowton, M. , Perez‐Cervantes, C. , Hur, S. , Jacobs‐Li, J. , Lu, E. , Deng, N. , Guzzetta, A. , Hoffmann, A. , Stocker, M. , Steimle, J. , Lazarevic, S. , Oubaha, S. , Yang, X. , Kim, C. , Yu, S. , Eckart, H. , Koska, M. , Hanson, E. , Chan, S. , … Moskowitz, I. (2022). Hedgehog signaling activates a mammalian heterochronic gene regulatory network controlling differentiation timing across lineages. Developmental Cell, 57, 2181–2203. 36108627 10.1016/j.devcel.2022.08.009 PMC10506397

[dgd12955-bib-0039] Saga, Y. , Hata, N. , Kobayashi, S. , Magnuson, T. , Seldin, M. F. , & Taketo, M. M. (1996). MesP1: a novel basic helix‐loop‐helix protein expressed in the nascent mesodermal cells during mouse gastrulation. Development, 122, 2769–2778. 8787751 10.1242/dev.122.9.2769

[dgd12955-bib-0040] Saga, Y. , Miyagawa‐Tomita, S. , Takagi, A. , Kitajima, S. , Miyazaki, J. , & Inoue, T. (1999). MesP1 is expressed in the heart precursor cells and required for the formation of a single heart tube. Development, 126, 3437–3447. 10393122 10.1242/dev.126.15.3437

[dgd12955-bib-0041] Shen, M. M. , Wang, H. , & Leder, P. (1997). A differential display strategy identifies cryptic, a novel EGF‐related gene expressed in the axial and lateral mesoderm during mouse gastrulation. Development, 124, 429–442. 9053319 10.1242/dev.124.2.429

[dgd12955-bib-0042] Tam, P. P. , Parameswaran, M. , Kinder, S. J. , & Weinberger, R. P. (1997). The allocation of epiblast cells to the embryonic heart and other mesodermal lineages: The role of ingression and tissue movement during gastrulation. Development, 124, 1631–1642. 9165112 10.1242/dev.124.9.1631

[dgd12955-bib-0043] Thomas, N. A. , Koudijs, M. , Van Eeden, F. J. , Joyner, A. L. , & Yelon, D. (2008). Hedgehog signaling plays a cell‐autonomous role in maximizing cardiac developmental potential. Development, 135, 3789–3799. 18842815 10.1242/dev.024083 PMC4213142

[dgd12955-bib-0044] Tickle, C. , & Towers, M. (2017). Sonic hedgehog signaling in limb development. Frontiers in Cell and Development Biology, 5, 14. 10.3389/fcell.2017.00014 PMC5328949 28293554

[dgd12955-bib-0045] Trapnell, C. , Cacchiarelli, D. , Grimsby, J. , Pokharel, P. , Li, S. , Morse, M. , Lennon, N. J. , Livak, K. J. , Mikkelsen, T. S. , & Rinn, J. L. (2014). The dynamics and regulators of cell fate decisions are revealed by pseudotemporal ordering of single cells. Nature Biotechnology, 32, 381–386. 10.1038/nbt.2859 PMC4122333 24658644

[dgd12955-bib-0046] Tsaytler, P. , Liu, J. , Blaess, G. , Schifferl, D. , Veenvliet, J. V. , Wittler, L. , Timmermann, B. , Herrmann, B. G. , & Koch, F. (2023). BMP4 triggers regulatory circuits specifying the cardiac mesoderm lineage. Development, 150, dev201450. 10.1242/dev.201450 PMC10233716 37082965

[dgd12955-bib-0047] Tsukui, T. , Capdevila, J. , Tamura, K. , Ruiz‐Lozano, P. , Rodriguez‐Esteban, C. , Yonei‐Tamura, S. , Magallón, J. , Chandraratna, R. A. S. , Chien, K. , Blumberg, B. , Evans, R. M. , & Belmonte, J. C. I. (1999). Multiple left‐right asymmetry defects in shh(−/−) mutant mice unveil a convergence of the shh and retinoic acid pathways in the control of Lefty‐1. Proceedings of the National Academy of Sciences of the United States of America, 96, 11376–11381. 10500184 10.1073/pnas.96.20.11376 PMC18041

[dgd12955-bib-0048] Tzahor, E. , & Evans, S. M. (2011). Pharyngeal mesoderm development during embryogenesis: Implications for both heart and head myogenesis. Cardiovascular Research, 91, 196–202. 21498416 10.1093/cvr/cvr116 PMC3125075

[dgd12955-bib-0049] Uehara, M. , Yashiro, K. , Takaoka, K. , Yamamoto, M. , & Hamada, H. (2009). Removal of maternal retinoic acid by embryonic CYP26 is required for correct nodal expression during early embryonic patterning. Genes & Development, 23, 1689–1698. 19605690 10.1101/gad.1776209 PMC2714714

[dgd12955-bib-0050] Wada, T. , Honda, M. , Minami, I. , Tooi, N. , Amagai, Y. , Nakatsuji, N. , & Aiba, K. (2009). Highly efficient differentiation and enrichment of spinal motor neurons derived from human and monkey embryonic stem cells. PLoS One, 4, e6722. 19701462 10.1371/journal.pone.0006722 PMC2726947

[dgd12955-bib-0051] Wong, K. S. , Rehn, K. , Palencia‐Desai, S. , Kohli, V. , Hunter, W. , Uhl, J. D. , Rost, M. S. , & Sumanas, S. (2012). Hedgehog signaling is required for differentiation of endocardial progenitors in zebrafish. Developmental Biology, 361, 377–391. 22119054 10.1016/j.ydbio.2011.11.004

[dgd12955-bib-0052] Yamaguchi, T. P. , Dumont, D. J. , Conlon, R. A. , Breitman, M. L. , & Rossant, J. (1993). Flk‐1, an flt‐related receptor tyrosine kinase is an early marker for endothelial cell precursors. Development, 118, 489–498. 8223275 10.1242/dev.118.2.489

[dgd12955-bib-0053] Yamamoto, M. , Mine, N. , Mochida, K. , Sakai, Y. , Saijoh, Y. , Meno, C. , & Hamada, H. (2003). Nodal signaling induces the midline barrier by activating nodal expression in the lateral plate. Development, 130, 1795–1804. 12642485 10.1242/dev.00408

[dgd12955-bib-0054] Zhang, X. M. , Ramalho‐Santos, M. , & Mcmahon, A. P. (2001). Smoothened mutants reveal redundant roles for shh and Ihh signaling including regulation of L/R symmetry by the mouse node. Cell, 106, 781–792. 11517919

[dgd12955-bib-0055] Zhou, L. , Liu, J. , Xiang, M. , Olson, P. , Guzzetta, A. , Zhang, K. , Moskowitz, I. P. , & Xie, L. (2017). Gata4 potentiates second heart field proliferation and hedgehog signaling for cardiac septation. Proceedings of the National Academy of Sciences of the United States of America, 114, E1422–e1431. 28167794 10.1073/pnas.1605137114 PMC5338429

