# Supplementary figures and images for: Sonic Hedgehog signaling regulates the optimal differentiation pace from early‐stage mesoderm to cardiogenic mesoderm in mice
Source: Dev Growth Differ. 2025 Jan 9;67(2):75–84. doi: 10.1111/dgd.12955 (PMC11842887; doi:10.1111/dgd.12955)

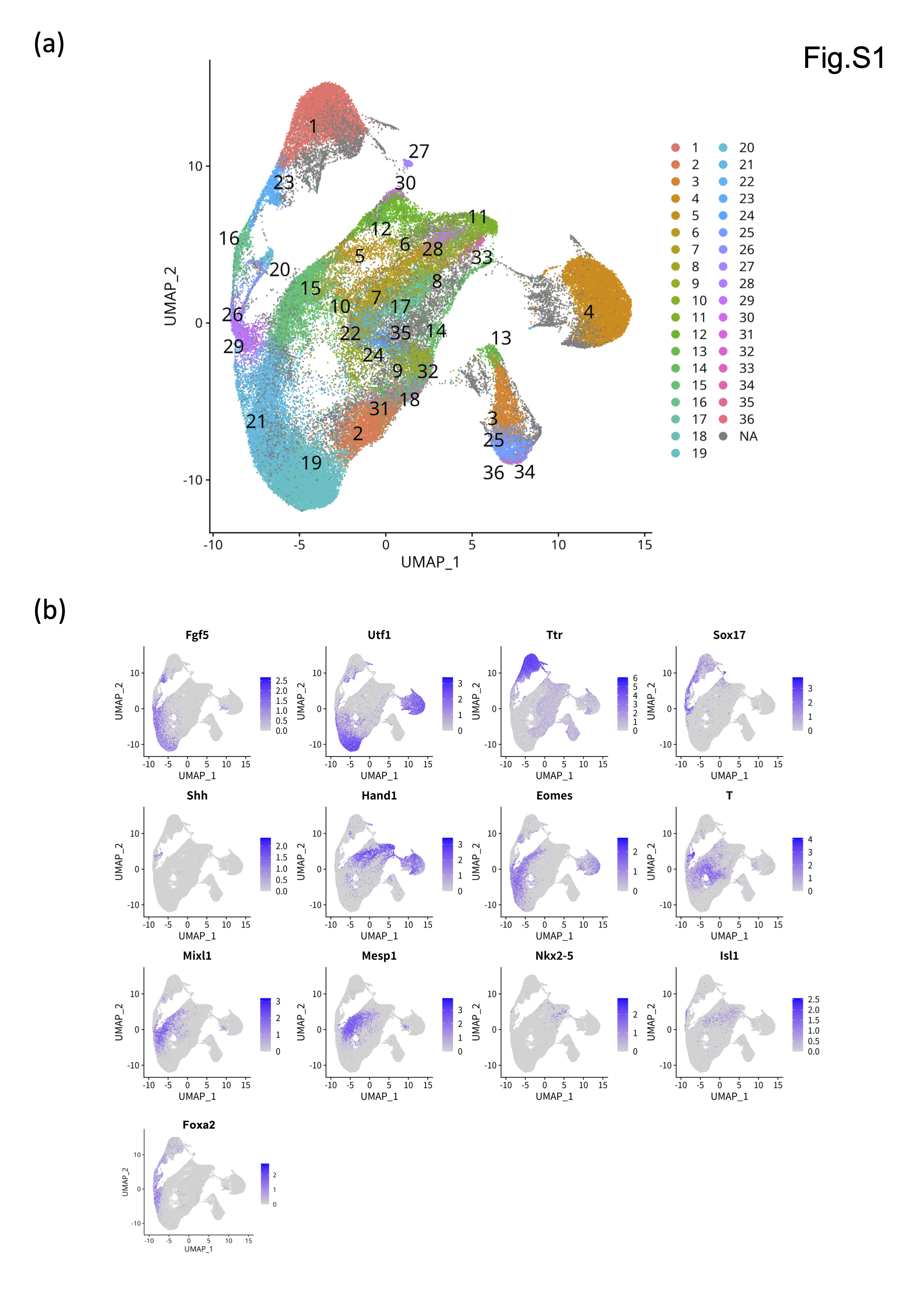

Supplement: Supplementary file 1 — Figure S1. Meta‐analysis of single‐cell (sc) RNA‐seq data from mouse embryos spanning the gastrula to the Early Headfold stage. (a) UMAP visualization of scRNA‐seq data, originally generated by Pijuan‐Sala et al. and deposited under accession number E‐MTAB‐6967 (Pijuan‐Sala et al., 2019). UMAP identified 37 clusters, each annotated according to previous report (Hao et al., 2024; Pijuan‐Sala et al., 2019). Notably, the T +/Foxa1 + cluster, originally annotated as “notochord”, reinterpreted as axial mesoderm expressing Shh. Additionally, we identified “mixed mesoderm” population as an intermediate state in the differentiation from Mesp1 + cardiogenic nascent mesoderm to lateral plate mesoderm and/or extraembryonic mesoderm. The clusters of “Primitive streak (cluster 21 in Figure S1)”, “Anterior primitive streak (cluster 29)”, “Notochord (cluster 20)”, “Nascent mesoderm (cluster 15)”, “Mixed mesoderm (cluster 5)”, “Extraembryonic mesoderm (cluster 6)”, “Pharyngeal mesoderm (cluster 8)”, and “Cardiomyocytes (cluster 33)” were subjected to sub‐clustering shown in Figure 1. Each cluster was annotated as followings according to the reference study: 1, Extraembryonic endoderm; 2, Rostral neuroectoderm; 3, Blood progenitors 2; 4, Extraembryonic ectoderm; 5, Mixed mesoderm; 6, Extraembryonic mesoderm; 7, Intermediate mesoderm; 8, Pharyngeal mesoderm; 9, Caudal epiblast; 10, Primordial germ cells; 11, Mesenchyme; 12, Hematoendothelial progenitors; 13, Blood progenitors 1; 14, Surface ectoderm; 15, Nascent mesoderm; 16, Gut; 17, Paraxial mesoderm; 18, Caudal neuroectoderm; 19, Epiblast; 20, Notochord; 21, Primitive streak; 22, Somitic mesoderm; 23, Visceral endoderm; 24, Caudal mesoderm；25, Erythroid 1; 26, Definitive endoderm; 27, Parietal endoderm; 28, Allantois; 29, Anterior primitive streak; 30, Endothelium; 31, Forebrain/Midbrain/Hindbrain; 32, Spinal cord; 33, Cardiomyocytes; 34, Erythroid 2; 35, Neuromesodermal progenitors; 36, Erythroid 3. Further details are provided i [file DGD-67-75-s001.tiff]

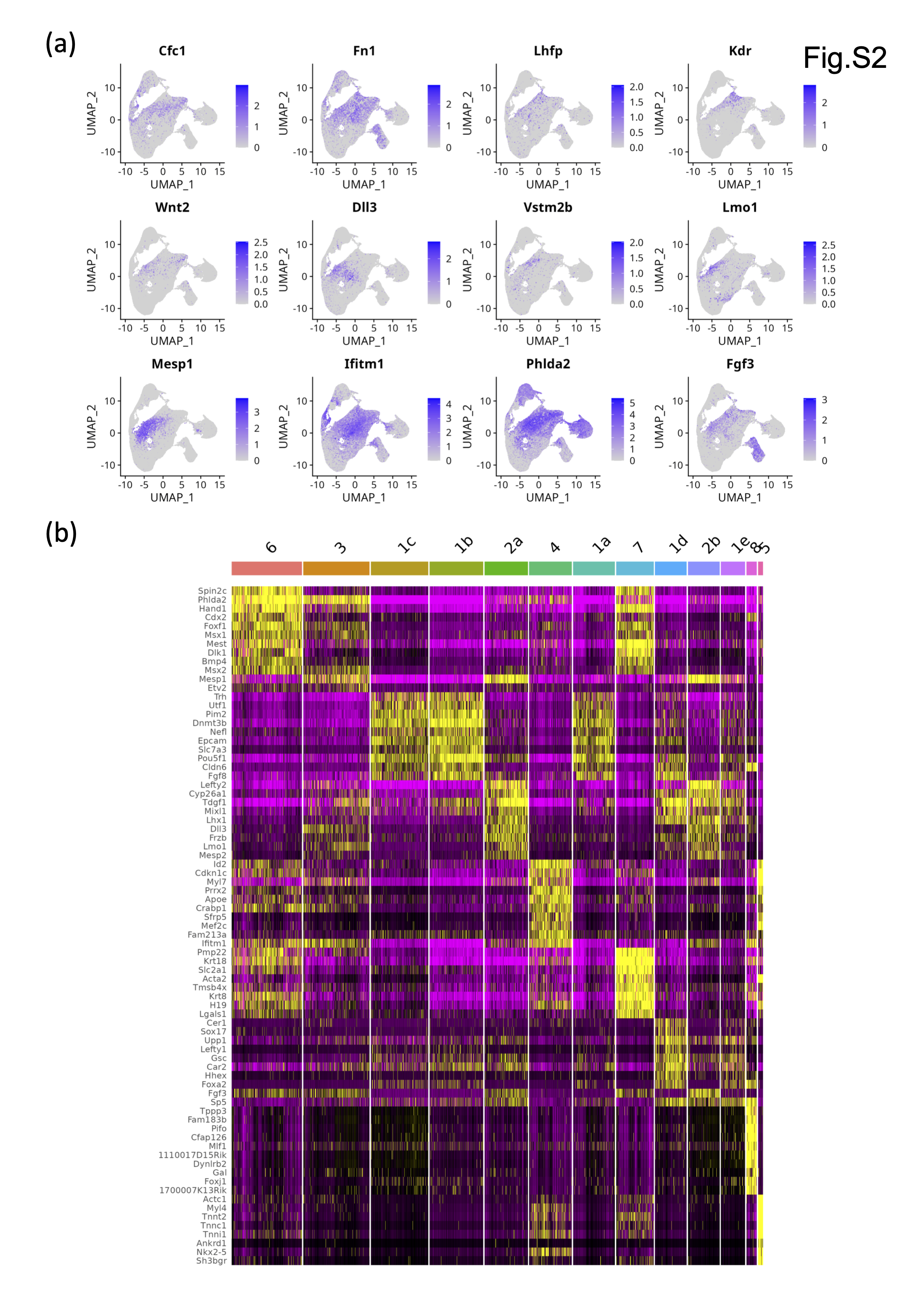

Supplement: Supplementary file 2 — Figure S2. Features of “mixed mesoderm”. (a) Genes expressed in “mixed mesoderm” cluster in Figure S1a. Note that mixed mesoderm expressed Cfc1 (Cryptic), Kdr, and Mesp1, representing that it is differentiating lateral plate mesoderm cells. (b) The principal marker genes used to annotate each cluster in sub‐clustering represented in Figure 1a. Based on these markers, clusters 1a, 1b, 1c, 1d, and 1e in Figure 1a were identified as the “primitive streak” and “anterior primitive streak”. Clusters 2a and 2b were classified as “nascent mesoderm”, cluster 3 as “mixed mesoderm”, cluster 4 as “pharyngeal mesoderm”, cluster 5 as “cardiomyocytes”, clusters 6 and 7 as “extraembryonic mesoderm”, and cluster 8 as “notochord”. The markers Fgf8, Mesp1, Kdr/Mesp1, Id2/Mef2, Nkx2‐5, Hand1, and Foxa2 identify the “primitive streak”. “nascent mesoderm”. “mixed mesoderm”. “pharyngeal mesoderm”. “cardiomyocytes”. “extraembryonic mesoderm”. and “notochord”. respectively. [file DGD-67-75-s003.tiff]

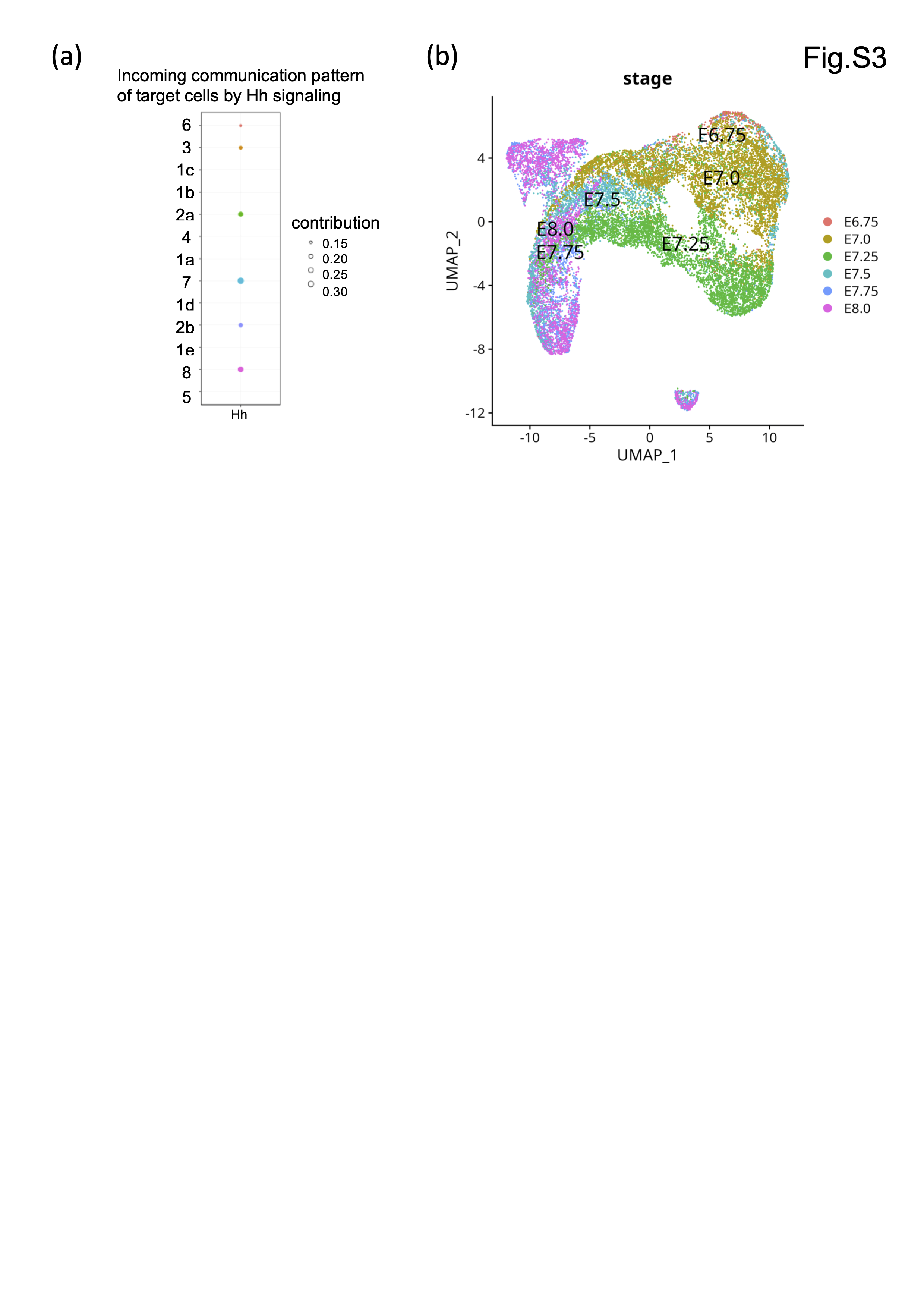

Supplement: Supplementary file 3 — Figure S3. Supporting data related to Figure 1. (a) Intercellular communication analysis was performed on the sub‐dataset using CellChat, as shown in Figure 1c. The CellChat analysis revealed that cardiac cells (clusters 2a, 2b, 3, and 4), which are positive for Mesp1, interact with the notochord (axial mesoderm) via Hedgehog (Hh) signaling. Refer to the main text for further details. (b) The embryonic stages corresponding to the clusters represented in Figure 1a, e are noted. The identification of two major primitive streak clusters is likely due to differences in the developmental stages. [file DGD-67-75-s005.tiff]

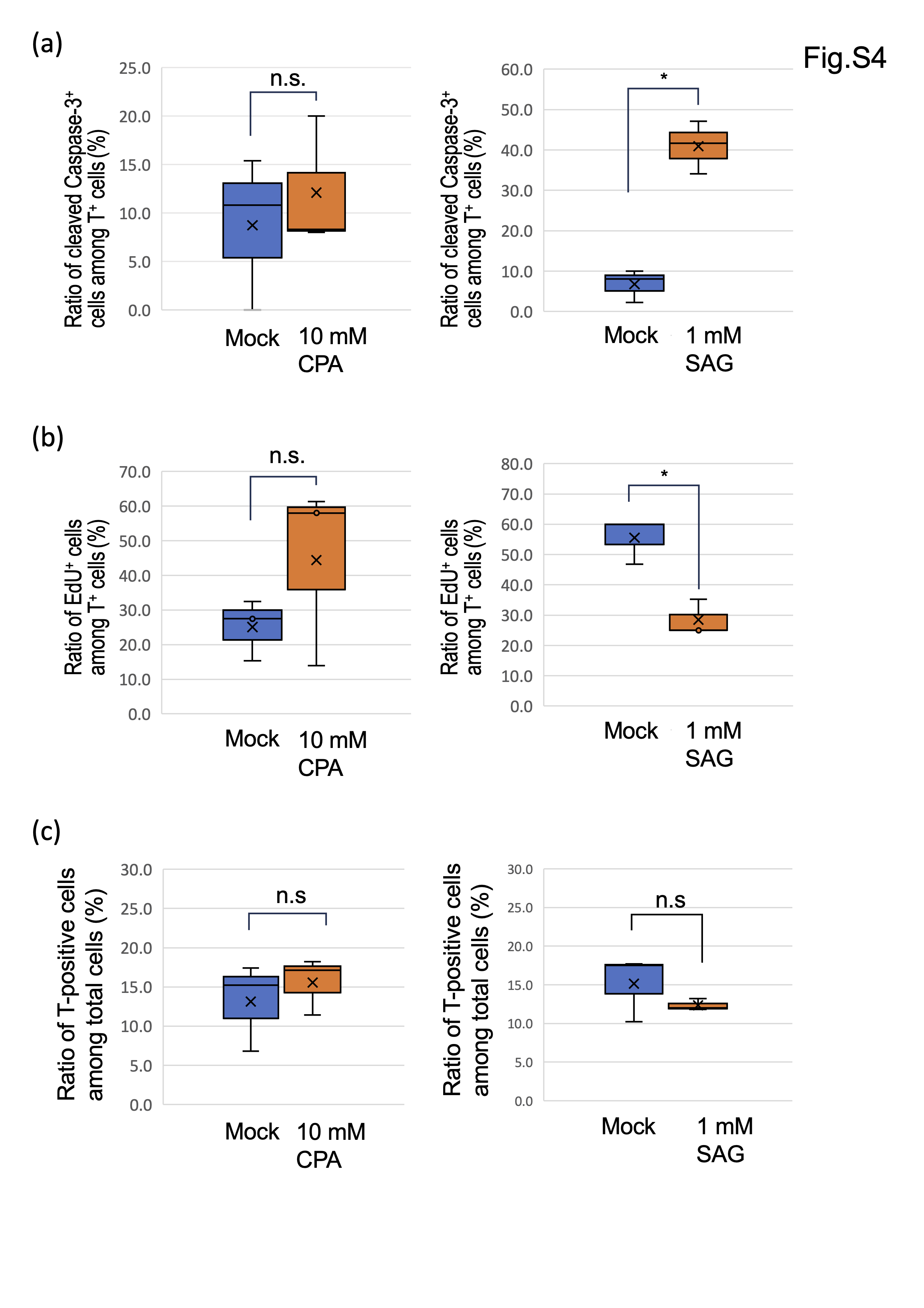

Supplement: Supplementary file 4 — Figure S4. The number of T‐expressing cells is not significantly altered by CPA or SAG treatment. (a) The ratio of apoptotic cells among T‐positive cells. The proportion of apoptotic cells among T‐positive cells differentiated from mouse ES cells was evaluated using immunofluorescence staining for cleaved Caspase‐3 and T on differentiation day 4, as presented in the box plot. These results are based on biological triplicate experiments. CPA treatment did not affect the proportion of apoptotic cells. In contrast, SAG treatment significantly increased apoptosis, despite a marked increase in T expression observed both in vitro and ex vivo with SAG treatment (Figures 2 and 3). Asterisk indicates statistical significance as p < .05. n.s., not significant. (b) The ratio of proliferative cells among T‐positive cells. The proportion of proliferative cells among T‐positive cells from mouse ES cells was assessed using the EdU incorporation assay and immunofluorescence staining for T on differentiation day 4, as shown in the box plot. These findings are based on results from biological triplicate experiments. CPA treatment did not significantly affect the proportion of proliferative cells. Conversely, SAG treatment markedly reduced the proportion of proliferative cells, despite a significant increase in T expression observed both in vitro and ex vivo with SAG treatment (Figures 2 and 3). Asterisk indicates statistical significance as p < .05. n.s., not significant. (c) The ratio of T‐positive cells among all cells. The proportion of T‐positive cells among the total cell population was assessed via immunofluorescence staining for T on differentiation day 4 and is depicted as a box plot. Notably, SAG treatment did not significantly affect the proportion of T‐positive cells, despite inducing apoptosis (a) and suppressing proliferation (b). While the inhibition of differentiation could potentially increase the number of T‐positive cells, the observed induction of apoptosis and sup [file DGD-67-75-s007.tiff]

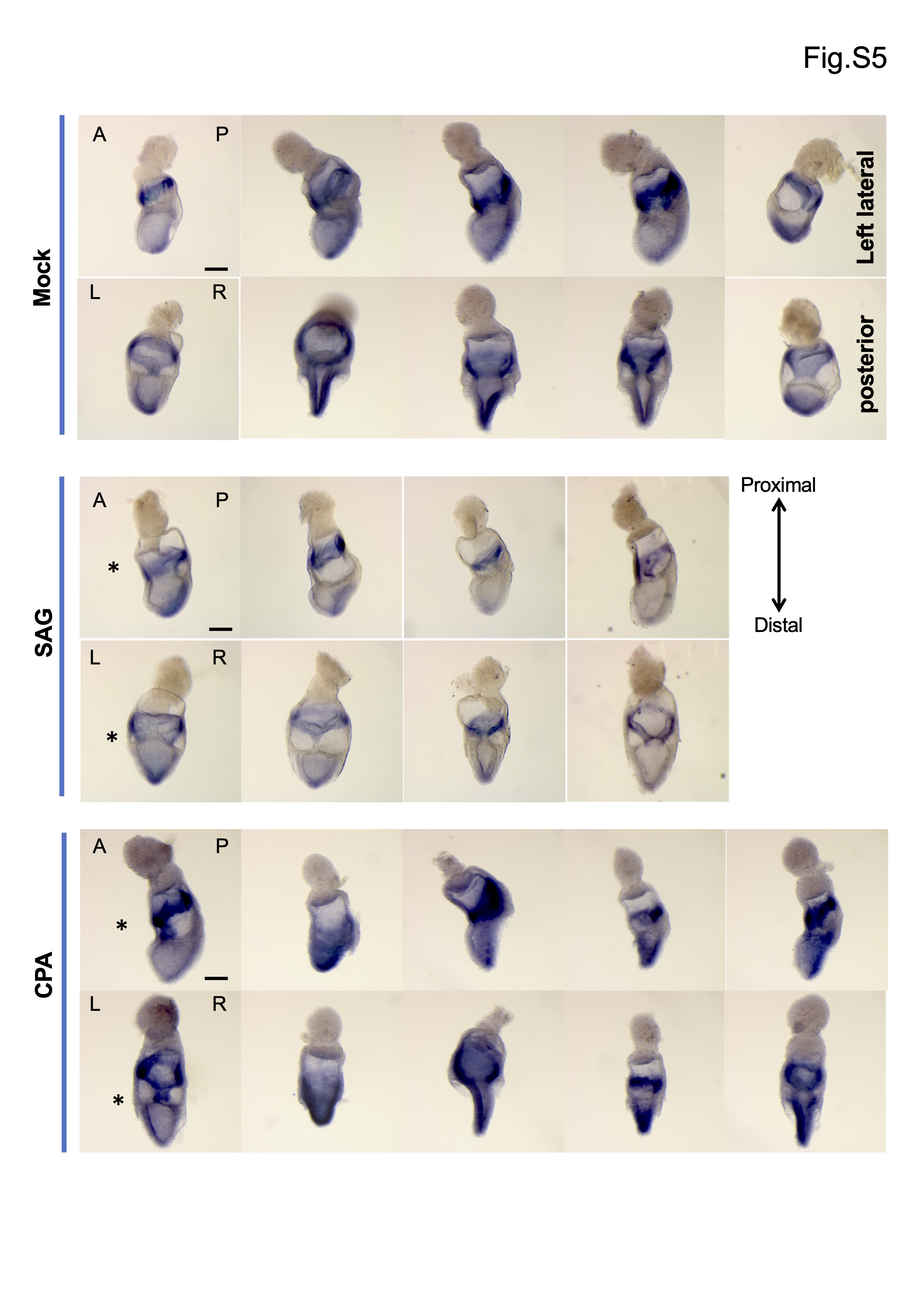

Supplement: Supplementary file 5 — Figure S5. The expression of Eomes in the embryos treated by SAG or CPA, not shown in Figure 3. For each group, upper column shows left lateral view, whereas the lower column shows posterior view. Data from the remaining five control embryos, four embryos treated with SAG, and five embryos treated with CPA, are presented here. Interestingly, the expression of Eomes in the extraembryonic tissue (Hancock et al., 1999) seemed to be not affected by altered Shh signaling. Asterisks indicate embryos with Eomes expression levels comparable to those of the control group. Scale bar, 250 μm. [file DGD-67-75-s006.tiff]

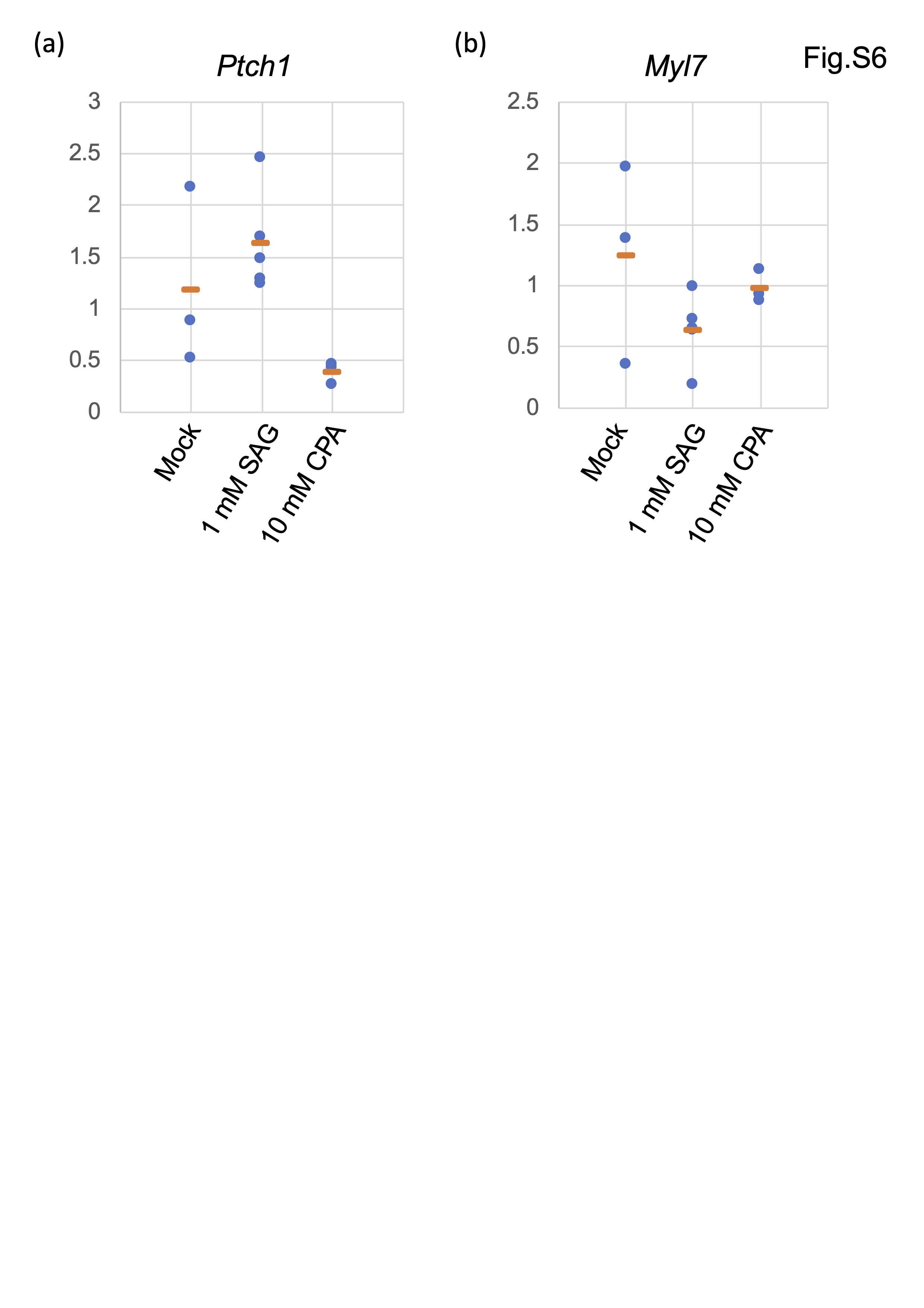

Supplement: Supplementary file 6 — Figure S6. The effect of SAG and CPA on Shh signal and cardiac differentiation in ex vivo embryos. The orange bar represents the median value. Embryos cultured for 24 h with SAG or CPA treatment were analyzed by real‐time RT‐PCR to assess the expression of Ptch1, a marker of Shh signaling (a), and Myl7, a sarcomeric gene indicative of cardiomyocyte differentiation (b). For Ptch1, the data include biological triplicates for the Mock treatment, quintuplets for SAG, and triplicates for CPA. No statistically significant differences were observed in the expression levels of Ptch1 and Myl7 in both SAG and CPA treatments. However, Ptch1 expression exhibited a trend of upregulation with SAG treatment and downregulation with CPA treatment, suggesting that SAG activates Shh signaling, while CPA suppresses it ex vivo (a). For Myl7, SAG treatment showed a trend toward decreased expression (b). Unfortunately, premature differentiation by CPA or suppressed cardiac differentiation by SAG were not clearly confirmed in these experiments. The lack of significant differences may be attributed to signals from cardiac cells being obscured by the presence of numerous non‐cardiac cells in the embryos. Alternatively, this could result from the challenge of harvesting mouse embryos at precisely the same developmental stage, leading to discrepancies in developmental stages at the start and end of the culture, which could contribute to high individual variation in drug effects. [file DGD-67-75-s002.tiff]

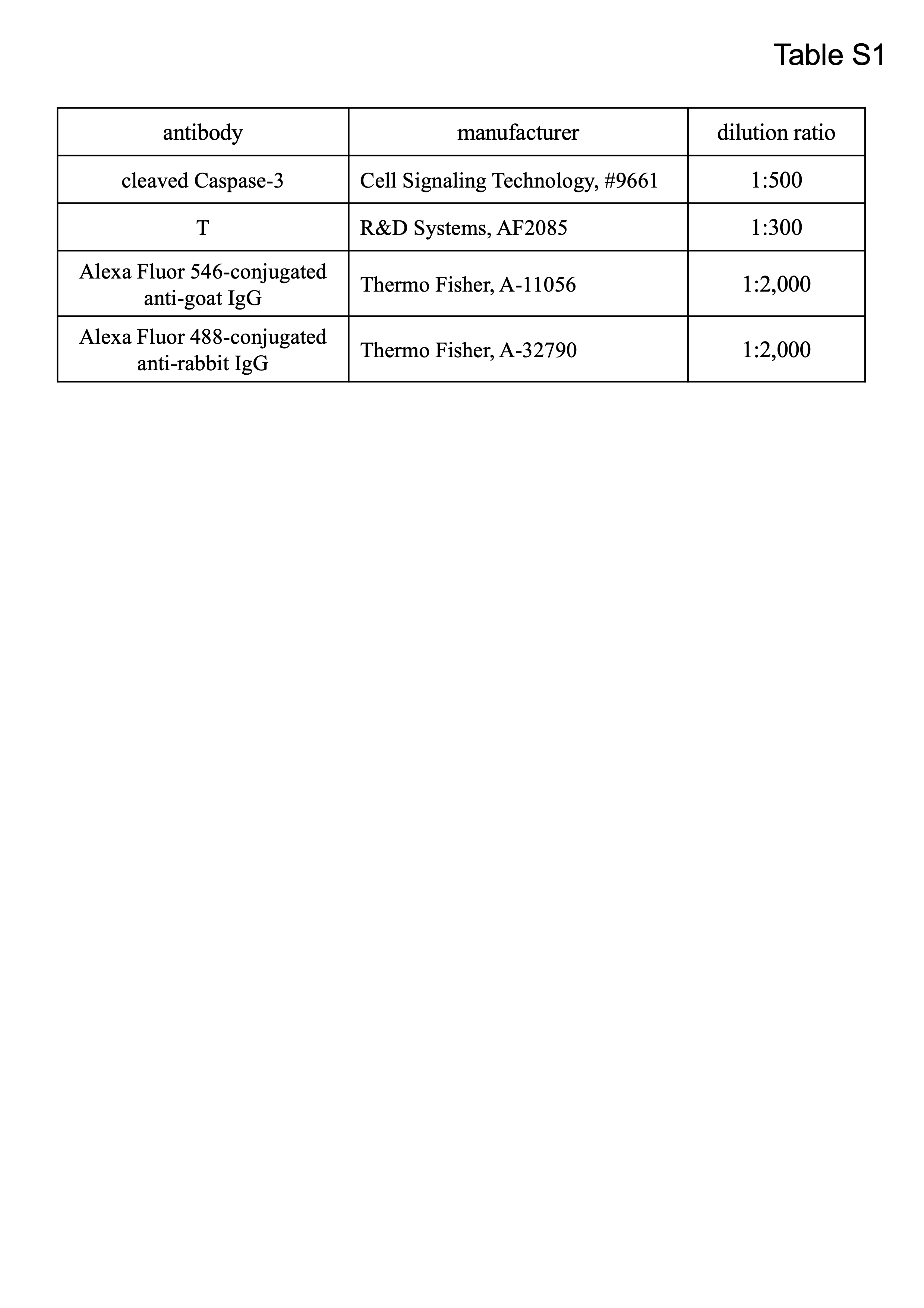

Supplement: Supplementary file 7 — Table S1. Used antibodies. [file DGD-67-75-s004.tiff]

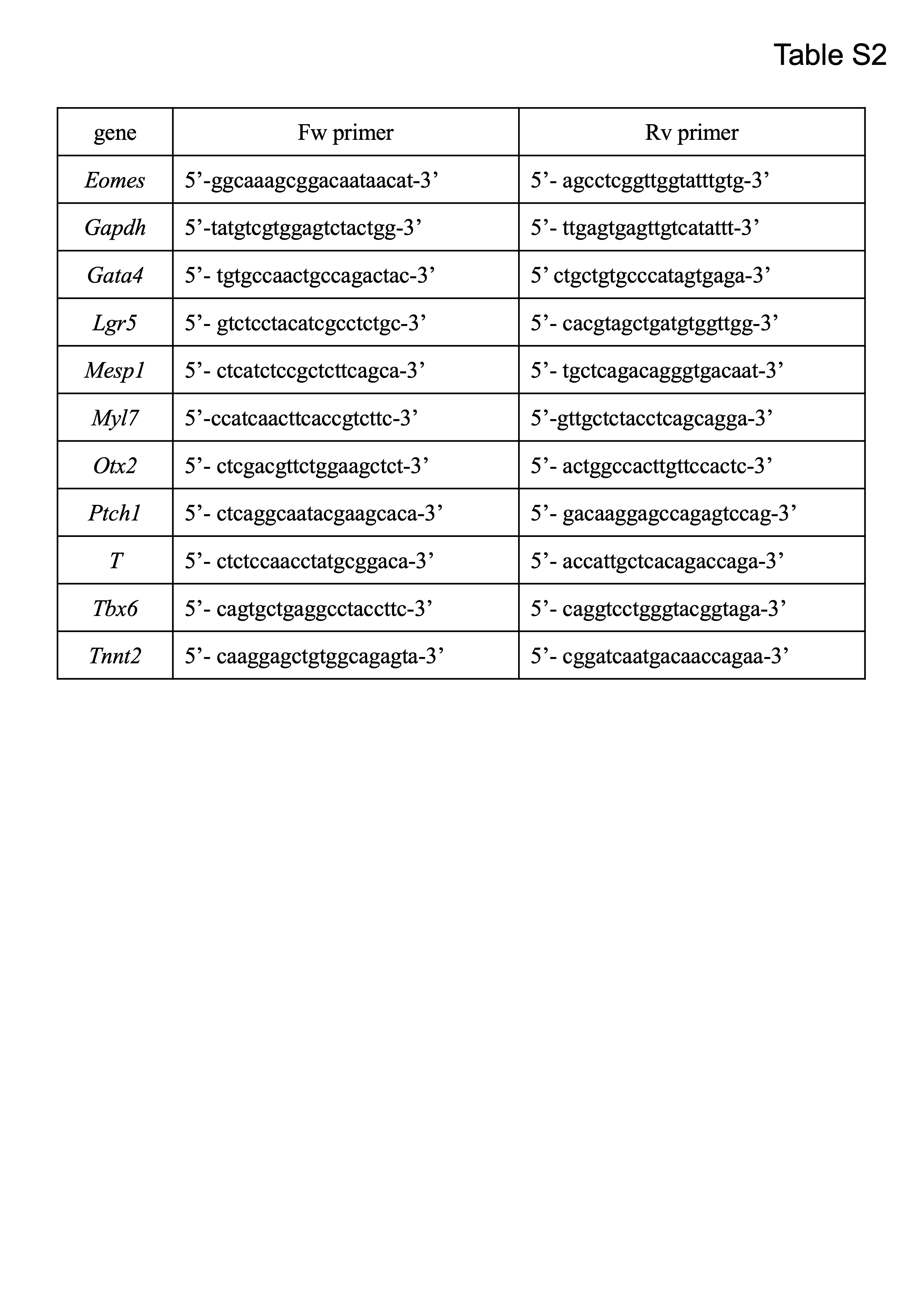

Supplement: Supplementary file 8 — Table S2. The used PCR primers. [file DGD-67-75-s008.tiff]
